# Comparisons between eyebags, droopy eyelids, and eyebrow positioning identified by photo‐numeric scales or identified by written descriptive scales: Insights from the Singapore/Malaysia cross‐sectional genetics epidemiology study (SMCGES) cohort

**DOI:** 10.1111/srt.13620

**Published:** 2024-02-20

**Authors:** Jun Yan Ng, Fook Tim Chew

**Affiliations:** ^1^ Department of Biological Sciences Faculty of Science National University of Singapore Singapore Singapore

**Keywords:** ageing, aging, anti‐ageing, droopy eyelids, eyebags, low eyebrow positioning, photo‐numeric scale, sagging, skin, written descriptive scale

## Abstract

**Background:**

We evaluate skin sagging phenotypes (eyebags, droopy eyelids, low eyebrow positioning) using written descriptive scales and photo‐numeric scales. We also study how anti‐ageing interventions and digital screen time influence skin sagging.

**Aim:**

We compare the two phenotype assessment methods with each other.

**Method:**

Skin sagging and personal lifestyle data obtained from 2885 ethnic Chinese young adults from the Singapore/Malaysia cross‐sectional genetics epidemiology study (SMCGES) cohort were collated and compared.

**Results:**

Significant correlations (*p*‐value < 0.001) between written descriptive scales and photo‐numeric scales were observed for eyebags (0.25) and eyebrow positioning (0.08). Significant correlations (*p*‐value < 0.001) were observed after combining both scales for eyebags (0.38), droopy eyelids (0.30), and eyebrow positioning (0.30). Anti‐ageing interventions are associated with delayed progression of eyebags from 18–45 years old, droopy eyelids from 31–45 years old, and eyebrow positioning from 35–40 years old. Significantly lower (*p*‐value < 0.02) eyebrow positioning is associated with both <1 and 1–3 h of screen time stratified by age.

**Conclusion:**

Written descriptive scales provide comparable results to photo‐numeric scales. However, validating and adapting photo‐numeric scales for different populations identifies phenotypes better. Anti‐ageing interventions are beneficial at different age ranges. Screen time is associated with skin sagging in young (18–30 years old) participants.

**Abbreviations**
AUC: area under curve
BMI: body mass index
cm: centimetres
IBM SPSS/PC: International Business Machines Corporation Statistical Package for Social Scientists Personal Computer (SPSS/PC)
ISAAC: International Study of Asthma and Allergies in Childhood
kg: kilograms
m2: squared metres
ROC: receiver operator characteristic
SD: standard deviation
SMCGES: Singapore/Malaysia Cross‐sectional Genetics Epidemiology Study

## 1 INTRODUCTION

Skin ageing phenotypes are commonly quantified using one of two methods: a written descriptive scale or a photo‐numeric scale. Both methods have their own strengths and limitations. In this study, our main objective is to compare the performance of written descriptive scales and photo‐numeric scales in assessing self‐reported skin ageing phenotypes.

Written descriptive scales consist of short and direct questions probing the presence or absence of a named phenotype.^1^, ^2^ Some questions yield a dichotomous response (e.g., yes or no) while other questions may be answered by selecting one of many options. Each option describes a different level of severity of the same phenotype.^3^ As they are short and concise, written descriptive scale questions are easy to deploy and replicate in questionnaires.

Photo‐numeric scales have been studied in detail in our previous studies.^4^, ^5^, ^6^ These scales consist of a set of photographs studying a specific phenotype (e.g., eyebags). The first photograph (i.e., photo 0) shows the absence of the phenotype (i.e., no eyebags). Subsequent photographs (i.e., photo 1, photo 2, etc.) show an increased presence of the phenotype of interest.

The written descriptive scale allows a phenotype to be assessed in a simple and direct way. However, it is highly reliant on using descriptive words to convey the intent of the questions. This leads us to hypothesise that phenotypes which are easy to describe in words perform better on a written descriptive scale as compared to phenotypes which are difficult to describe in words. In contrast, photo‐numeric scales do not share this limitation because they describe a phenotype through photographs. Instead, photo‐numeric scales face other challenges as the same phenotype may present itself differently on the skin of different genders or ethnicities. These challenges are separately discussed in our previous studies.^4^, ^5^

Here, we investigate how well the written descriptive scale and photo‐numeric scale perform in evaluating the same phenotype. We have identified three skin sagging phenotypes which are easy to describe in words: they are eyebags (i.e., skin below the eyes sagging downwards), droopy eyelids (i.e., eyelids which sag downwards), and low eyebrow positioning (i.e., eyebrows sagging close to the eyes). These three phenotypes are selected because firstly, they are concentrated in roughly the same area of the face, secondly, they are in a highly viewed location, and thirdly, they can be described using commonly used words. We believe that these characteristics enable participants to have a keen and subconscious awareness of their face such that any subtle changes in these aspects of their faces will be noticed in a matter of days. As such, participants can provide very up‐to‐date information on the state of their skin in these three areas.

Based on our hypothesis, participants can intuitively understand what eyebags, droopy eyelids, and low eyebrows are, because these are simple terms. Participants can also readily identify the presence or absence of eyebags, droopy eyelids, and low eyebrows on their faces with the subconscious and up‐to‐date information which they possess. Therefore, participants who self‐report the absence of these skin sagging phenotypes on the written descriptive scale will also choose photographs showing the absence of these respective phenotypes (i.e., photo 0). Likewise, participants who self‐report the presence of these skin sagging phenotypes on the written descriptive scale will also choose photographs showing the presence of these respective phenotypes (i.e., photos 1 and above).

In this study, we compare the self‐reported evaluation of eyebags, droopy eyelids, and low eyebrows on a written descriptive scale and on a photo‐numeric scale. Using the results of these comparisons, we evaluate the performance of these two commonly used methods to quantify skin ageing phenotypes.

## 2 METHODS

### 2.1 Participant recruitment

Recruitment was conducted from the Singapore/Malaysia Cross‐sectional Genetics Epidemiology Study (SMCGES) cohort for this skin ageing study. The SMCGES cohort consists of participants from Singapore and Malaysia and has been previously studied in epidemiological and genetic studies of allergic diseases. We have previously described this cohort in detail elsewhere.^7^, ^8^, ^9^

Participants for the previous study (i.e., the epidemiological and genetic study) were recruited via emails and posters from the National University of Singapore, Singapore (2005–2023), Universiti Tunku Abdul Rahman (UTAR) Campus, Malaysia (2016–2018), and Sunway University, Malaysia (2019 and 2022). These participants walked in and voluntarily joined our study.

Some participants consent to be re‐contacted. We invited these participants to participate in this present study (i.e., the skin ageing study). A total of 10,248 participants were invited to participate in the skin ageing study, of which 3365 completed the study. The demographics of the respondents and non‐respondents are similar and can be found elsewhere.^5^ All 3365 participants constitute our study population. From this study population, we drew a subset of 2885 ethnic Chinese young adult participants (Table 1).

**TABLE 1:** Summary table for demographics drawn from a population of young Singapore and Malaysia adults recruited from the Singapore/Malaysia cross‐sectional genetics epidemiology study (SMCGES) cohort.

Demographic factor	All respondents	Chinese respondents
**Participants**	3365 (100%)	2885 (100%)
**Mean age (years) ± SD**	26.3 ± 6.9	26.2 ± 6.6
**Mean height (cm) ± SD**	165.4 ± 8.7	165.6 ± 8.4
**Mean weight (kg) ± SD**	60.9 ± 13.3	60.1 ± 12.7
**Age range (years)**	18 to 73	18–73
**BMI (kg/m^2^) ± SD**	22.2 ± 4.9	21.8 ± 3.7
**Gender**		
Male	1227 (36.5%)	1053 (36.5%)
Female	2138 (63.5%)	1832 (63.5%)
**Ethnicity**		
Chinese	2885 (85.7%)	2885 (100%)
**Total monthly family income per capita**		
Low	386 (11.5%)	302 (10.5%)
Moderate	774 (23.0%)	650 (22.5%)
High	792 (23.5%)	682 (23.6%)
Very high	1396 (41.5%)	1234 (42.8%)
Missing/Invalid	17 (0.5%)	17 (0.6%)
**Housing**		
Flat	1794 (53.3%)	1590 (55.1%)
Condominium/Private apartment	852 (25.3%)	695 (24.1%)
Landed property	698 (20.7%)	579 (20.1%)
Missing/Invalid	21 (0.6%)	21 (0.7%)
**Usage of anti‐ageing skincare products**		
Yes	479 (14.2%)	420 (14.6%)
No	2886 (85.8%)	2465 (85.4%)
**Hours spent daily in front of the television or computer**		
Less than 1 h	75 (2.2%)	65 (2.3%)
1–3 h	560 (16.6%)	497 (17.2%)
More than 3–5 h	895 (26.6%)	778 (27.0%)
More than 5 h	1835 (54.5%)	1545 (53.6%)

The values after ± are standard deviation values. Missing/Invalid refers to responses that are either left blank or otherwise invalid.

Abbreviations: BMI, body mass index; cm, centimetres; kg, kilograms; m^2^, squared meters; SD, standard deviation.

The study was conducted in accordance with the Declaration of Helsinki and Good Clinical Practices.

### 2.2 Survey data collection

The SMCGES cohort participants have previously completed a set of investigator‐administered, validated International Study of Asthma and Allergies in Childhood (ISAAC) questionnaires. In these questionnaires, participants were asked to provide sociodemographic data, personal lifestyle data (e.g., usage of anti‐ageing skincare creams, substances, or therapies, digital screen time), and familial and personal medical history data.

A dichotomous question (i.e., ‘Have you ever applied any anti‐ageing creams, consumed any anti‐ageing substances or undergone any anti‐ageing therapies?) was used to study the usage of anti‐ageing skincare products.

To study digital screen time, participants were asked the following question: ‘How many hours do you spend in front of the television or computer every day?’. The collected data falls in one of four levels of exposure: less than 1 h, 1–3 h, more than 3–5 h, and more than 5 h. After completing the ISAAC questionnaires, most of the participants agreed to take part in a separate study on skin ageing (i.e., this present study). All the participants who agreed to join the skin ageing study signed an informed consent form. The skin ageing study is an investigator‐administered skin ageing questionnaire. In this questionnaire, participants self‐reported various skin ageing phenotypes on their skin; this will be elaborated in the next section. After completing the self‐evaluation, participants provided more data on their personal lifestyle.

### 2.3 Evaluating skin ageing

Skin ageing is self‐reported by the participant using (a) a written descriptive scale, and (b) a photo‐numeric scale. Three skin sagging phenotypes were evaluated in this way. They are eyebags (i.e., skin below the eyes sagging downwards), droopy eyelids (i.e., eyelids which sag downwards), and low eyebrow positioning (i.e., eyebrows sagging close to the eyes).

The self‐evaluation was conducted with the aid of a dressing table mirror. Each participant was provided with a personal dressing table mirror. All evaluations were performed in an indoor environment with standard lighting.

### 2.4 Statistical analysis

All the photo‐numeric scales are standardised.

Two‐tailed bivariate correlations for Spearman's Rank correlation (*ρ*) and Cohen's Kappa (*κ*) are calculated using Version 25 of the IBM Statistical Package for Social Scientists (SPSS/PC) and reported in Table 2. The strengths are interpreted as follows: 0.00–0.19: weak, 0.20–0.39: moderate, 0.40–0.59: fairly strong, 0.60–0.79: strong, and 0.80–1.00: very strong.

**TABLE 2:** Concordance, agreement, sensitivity, specificity, and area under curve (AUC) between the photo‐numeric scale and the written descriptive scale.

Phenotype	Eyebags				Droopy eyelids				Low eyebrow positioning			
Putative gold standard is the:	Photo‐numeric scale		Written descriptive scale		Photo‐numeric scale		Written descriptive scale		Photo‐numeric scale		Written descriptive scale	
Measurement	Value	*p*‐Value	Value	*p*‐Value	Value	*p*‐Value	Value	*p*‐Value	Value	*p*‐Value	Value	*p*‐Value
**Spearman's rank correlation (*ρ*)**	0.25	9.0E‐42	0.25	9.0E‐42	0.05	1.1E‐02	0.05	1.1E‐02	0.08	1.0E‐05	0.08	1.0E‐05
**Cohen's Kappa (*κ*)**	0.13	5.3E‐17	0.13	5.3E‐17	0.04	1.1E‐02	0.04	1.1E‐02	0.04	4.1E‐05	0.04	4.1E‐05
**Sensitivity**	0.60		0.89		0.21		0.10		0.07		0.63	
**Specificity**	0.61		0.23		0.86		0.94		0.97		0.54	
**Area under curve (AUC)**	0.44	7.1E‐08	0.64	1.2E‐36	0.54	9.8E‐02	0.52	2.6E‐01	0.51	4.7E‐01	0.60	6.3E‐05

Sensitivity and specificity are computed using SPSS. In calculating sensitivity and specificity, photo‐numeric scale measurements of photo 1 and above indicate presence of the phenotype. Photo 0 on the photo‐numeric scale indicates absence of the phenotype. The strengths are interpreted as follows: 0.00–0.19: low, 0.20–0.39: moderate, 0.40–0.59: fairly high, 0.60–0.79: high, and 0.80–1.00: very high.

The area under curve (AUC) of the Receiver Operator Characteristic (ROC) curve is computed using SPSS. AUC curves compare the grades for eyebags (Figure S1), droopy eyelids (Figure S1), and low eyebrow positioning (Figure S1) evaluated using a written descriptive scale or a photo‐numeric scale. The strength of the AUC is interpreted as follows: 0.51–0.59: weak, 0.60–0.69: moderate, 0.70–0.79: fairly strong, 0.80–0.89: strong, and 0.90–1.00: very strong. AUCs in the opposite direction (i.e., <0.50) are described in the same way (e.g., an AUC of 0.40–0.49 is also interpreted as weak).

Chi‐square tests are performed to evaluate the proportion of participants with eyebags, droopy eyelids, and eyebrow positioning of different severity levels. Chi‐square trend tests are performed to evaluate whether the changes in these proportions follow a significant trend with chronological age.

## 3 RESULTS

### 3.1 Participant demographics

We studied a group of 2,885 ethnic Chinese participants. They provide a representative overview of the epidemiology of skin ageing in the Singapore ethnic Chinese young adult population. This group consists of more females (*n* = 1832, 63.5%) than males. The average age of the participants is 26.2 ± 6.6 years old. Our study population consists predominantly of young adults aged 21–30. The youngest participant is 18 years’ old, and the oldest participant is 73 years’ old (Figure S2). Despite being a relatively young study cohort, we report that significant differences exist in the manifestation of skin ageing phenotypes around the eyes, particularly eyebags, within these young age groups (Chi‐square test *p*‐value < 0.001) (Figure S3a). Eyebags severity increases progressively with age (Chi‐square trend test *p*‐value < 0.001) (Figure S3a). Droopy eyelids steadily worsen with age (Chi‐square trend test *p*‐value < 0.001) (Figure S3b). We also report a significant trend in which eyebrow positioning becomes lower with age (Chi‐square trend test *p*‐value < 0.001) (Figure S3c). Our participants are 165.6 ± 8.4 cm in height, 60.1 ± 12.7 kg in weight, and have a BMI of 21.8 ± 3.7 kg/m^2^. Most participants have very high total monthly family income per capita (*n* = 1234, 42.8%) and most participants stay in flats (*n* = 1590, 55.1%). Most participants do not use anti‐ageing skincare products (*n* = 2465, 85.4%). Most participants spend more than 5 h in front of the television or computer every day (*n* = 1545, 53.6%), followed by ‘more than 3–5 h’ (*n* = 778, 27.0%), ‘1–3 h’ (*n* = 497, 17.2%), and a small number of participants (*n* = 65, 2.3%) spend less than 1 h in front of the television or computer every day (Table 1).

### 3.2 Studying self‐reported phenotypes

In our previous study, we discussed in detail the suitability of using self‐reported phenotypes in place of assessor‐evaluated phenotypes. We found that self‐reported phenotypes provide a satisfactory approximation of the phenotype scores given by our trained assessors.^6^ Hence, our study here focuses on evaluating self‐reported phenotypic data.

### 3.3 Eyebags

Eyebags evaluated using a photo‐numeric scale are moderately correlated with eyebags evaluated using a written descriptive scale (Spearman correlation = 0.25, *p*‐value < 0.001) (Table 2). The two scales significantly agree with each other (Cohen's Kappa = 0.13, *p*‐value < 0.001) (Table 2), but weakly. Sensitivity (0.60) and specificity (0.61) of using the photo‐numeric scale as the gold standard are both high. The area under curve (AUC) is significant (*p* < 0.001) (Table 2) in the opposite direction but weak (0.44). When the written descriptive scale is treated as the gold standard, sensitivity is very high (0.89), specificity is moderate (0.23) and the AUC is moderate (0.64) (Table 2).

**FIGURE 1:**
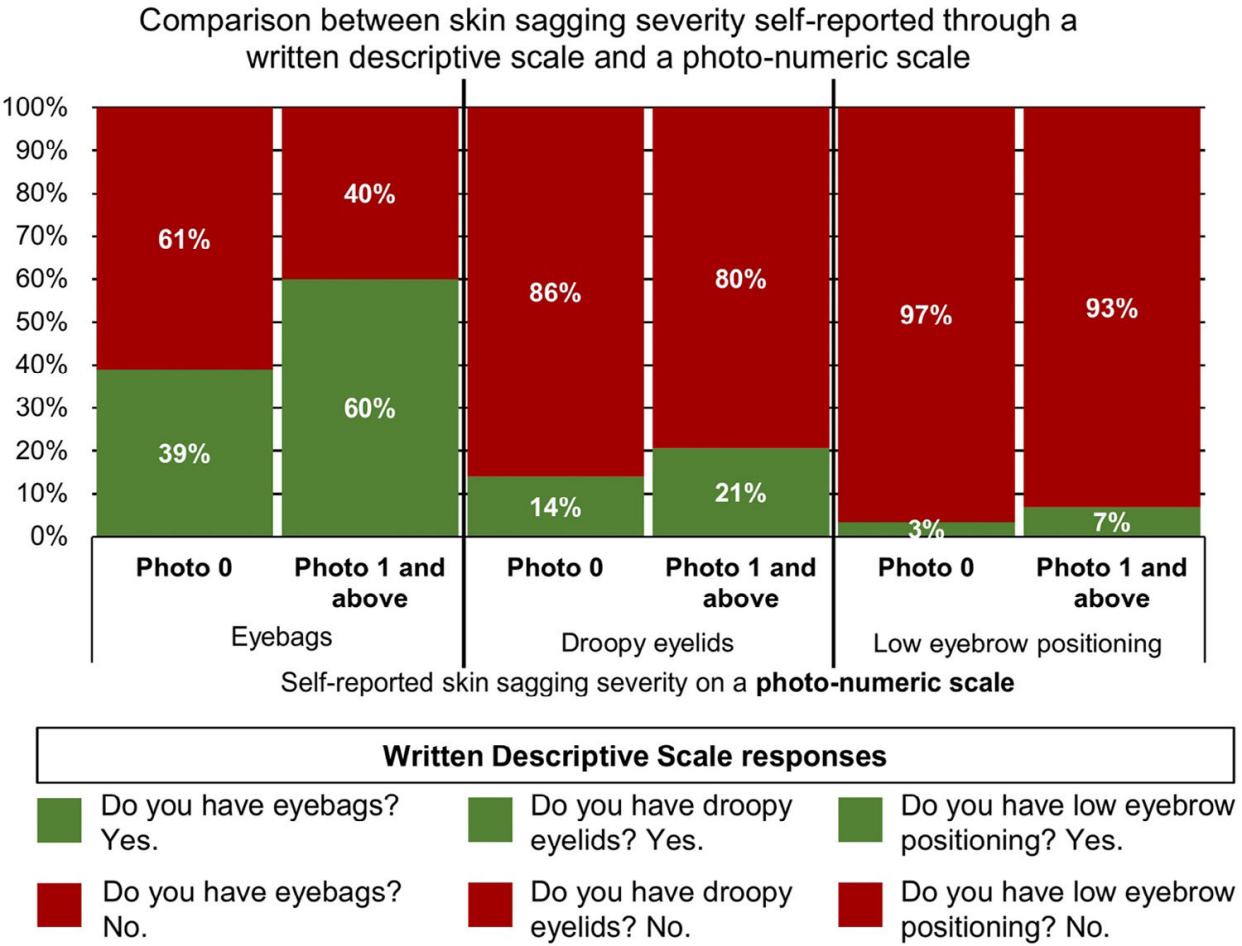
Comparison between self‐reported skin sagging on a written descriptive scale (e.g., Do you have eyebags? Do you have droopy eyelids? Do you have low eyebrow positioning?) and self‐reported eyebags, droopy eyelids, and low eyebrow positioning on their respective photo‐numeric scales.

**FIGURE 2:**
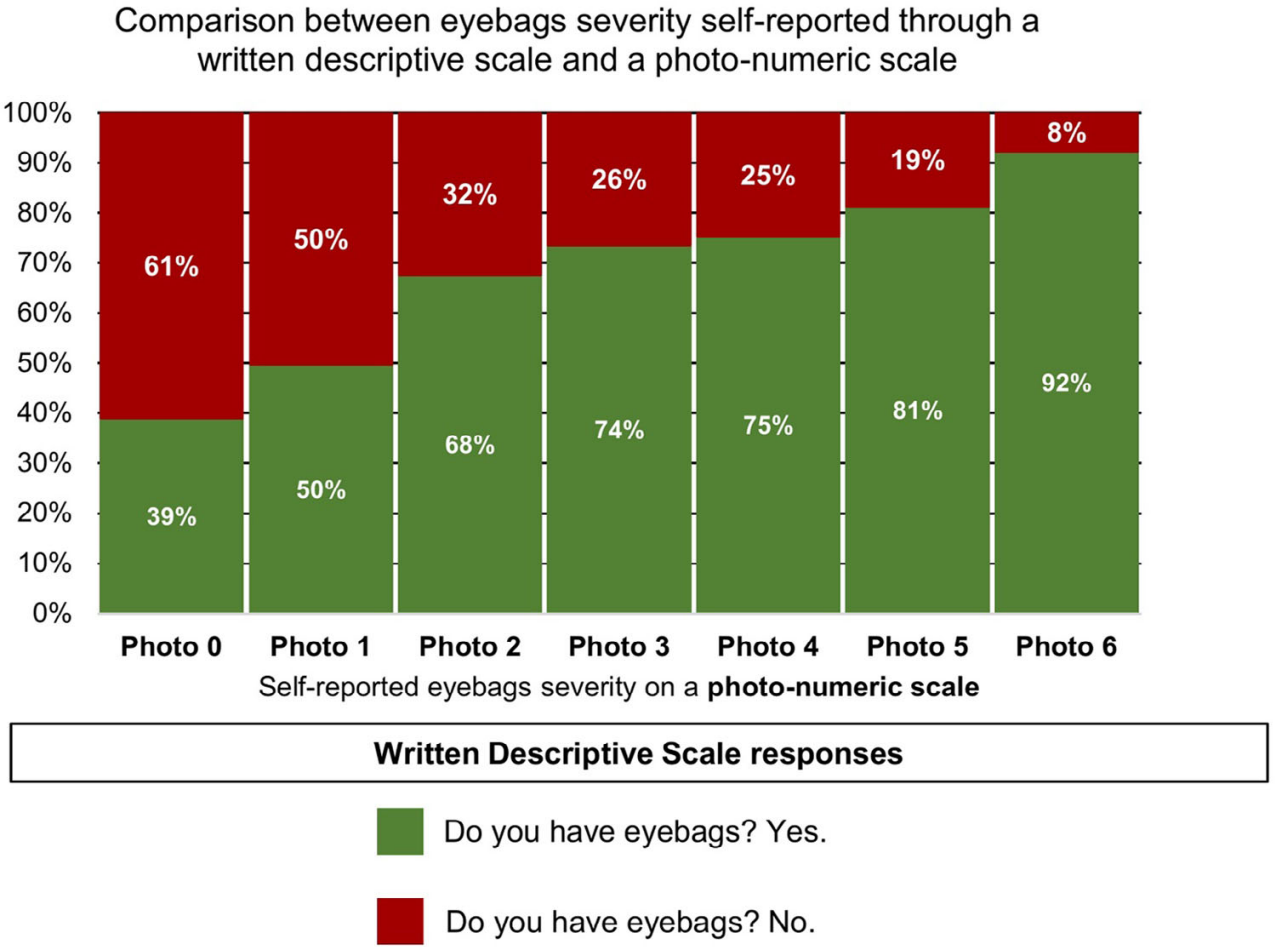
Comparison between self‐reported eyebags on a written descriptive scale (i.e., Do you have eyebags?) and self‐reported eyebags on a photo‐numeric scale. The photo‐numeric scale for eyebags consists of seven levels ranging from Photo 0 (no eyebags) to Photo 6 (severe eyebags).

Among participants evaluated to have no eyebags on the photo‐numeric scale (i.e., photo 0), a majority (61%) also reported no eyebags on the written descriptive scale (Figure 1). Among participants evaluated to have eyebags on the photo‐numeric scale (i.e., photo 1 and above), a majority (60%) also reported to have eyebags on the written descriptive scale (Figure 1). Eyebags severity can be further divided into six levels: mild eyebags (i.e., photos 1 and 2), moderate eyebags (i.e., photos 3 and 4), and severe eyebags (i.e., photos 5 and 6). About 50%–68% of participants who identified with mild eyebags (i.e., photos 1 and 2) also responded with a ‘yes’ to the question ‘Do you have eyebags?’ (Figure 2). This percentage increased to 74%–75% among participants who identified with moderate eyebags (i.e., photos 3 and 4). This percentage further increased to 81%–92% among participants who identified with severe eyebags (i.e., photos 5 and 6) (Figure 2).

### 3.4 Droopy eyelids

Droopy eyelids evaluated using a photo‐numeric scale are correlated with droopy eyelids evaluated using a written descriptive scale (Spearman correlation = 0.05, *p*‐value < 0.05) (Table 2), but weakly. The two scales significantly agree with each other (Cohen's Kappa = 0.04, *p*‐value < 0.05) (Table 2), but weakly. When the photo‐numeric scale is treated as the gold standard, sensitivity is moderate (0.21), specificity is very high (0.86), and the AUC is weak (0.54). When the written descriptive scale is treated as the gold standard, sensitivity is low (0.10), specificity is very high (0.94), and the AUC is weak (0.52) (Table 2).

**FIGURE 3:**
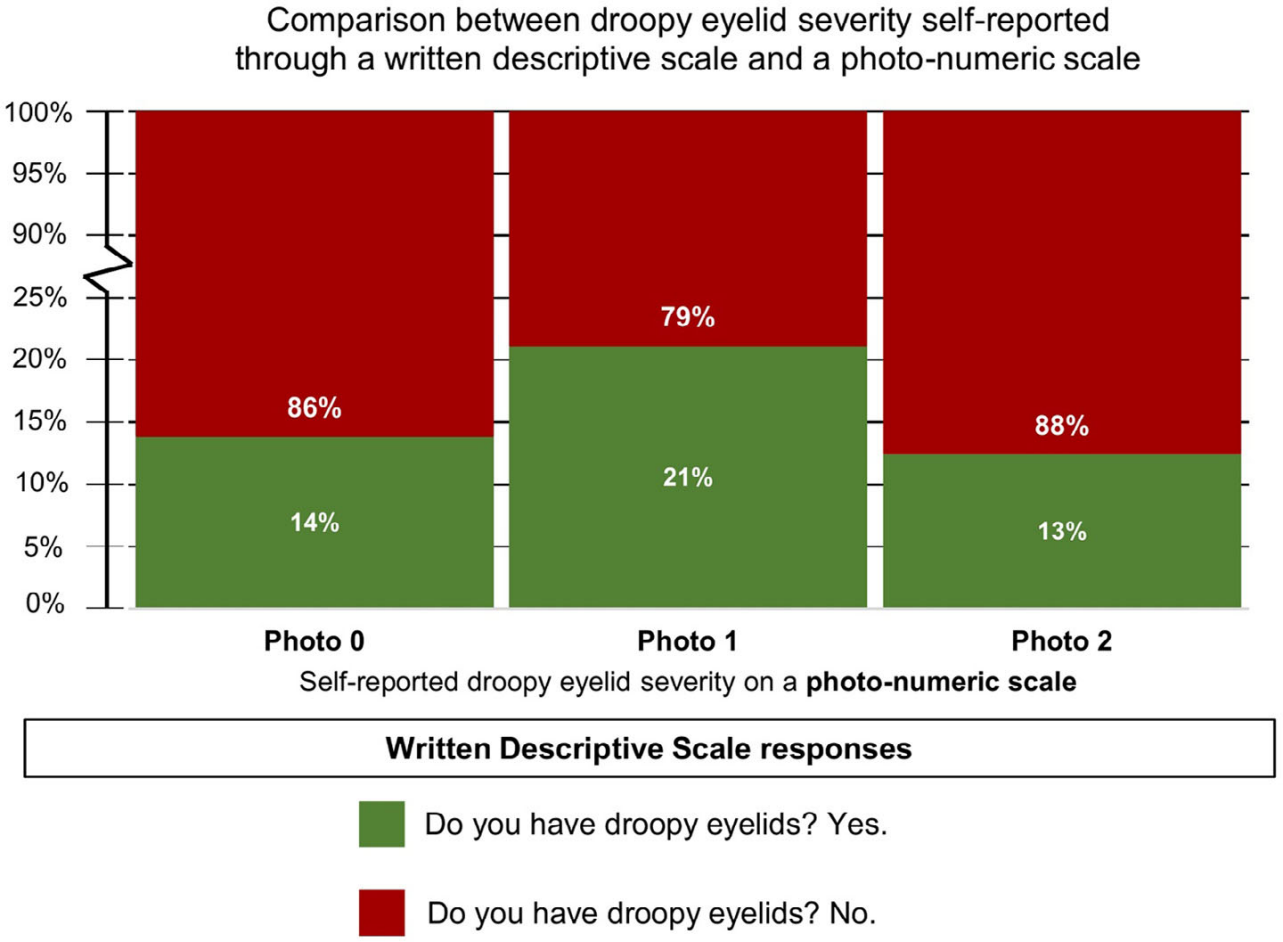
Comparison between self‐reported droopy eyelid severity on a written descriptive scale (i.e., Do you have droopy eyelids?) and self‐reported droopy eyelids on a photo‐numeric scale. The photo‐numeric scale for droopy eyelids consists of three levels ranging from Photo 0 (no droopy eyelids) to Photo 2 (severe droopy eyelids).

Among participants who self‐reported to have no droopy eyelids on the photo‐numeric scale (i.e., photo 0), a majority (86%) also reported no droopy eyelids on the written descriptive scale (Figure 1). Among participants who self‐reported to have droopy eyelids on the photo‐numeric scale (i.e., photo 1 and above), 21% of them also reported to have droopy eyelids on the written descriptive scale (Figure 1). Droopy eyelids on the photo‐numeric scale can be further divided into mild droopy eyelids (i.e., photo 1) and severe droopy eyelids (i.e., photo 2). About 21% of participants who identified with mild droopy eyelids (i.e., photo 1) also responded with a ‘yes’ to the question ‘Do you have droopy eyelids?’ (Figure 3). About 13% of participants who identified with severe droopy eyelids (i.e., photo 2) also responded with a ‘yes’ to the question ‘Do you have droopy eyelids’ (Figure 3).

### 3.5 Low eyebrow positioning

Low eyebrow positioning evaluated using a photo‐numeric scale are correlated with low eyebrow positioning evaluated using a written descriptive scale (Spearman correlation = 0.08, *p*‐value < 0.001) (Table 2), but weakly. The two scales significantly agree with each other (Cohen's Kappa = 0.04, *p*‐value < 0.001) (Table 2), but weakly. When the photo‐numeric scale is treated as the gold standard, sensitivity is low (0.07), specificity is very high (0.97), and the AUC is weak (0.51). When the written descriptive scale is treated as the gold standard, sensitivity is high (0.63), specificity is fairly high (0.54), and the AUC is moderate (0.60) (Table 2).

**FIGURE 4:**
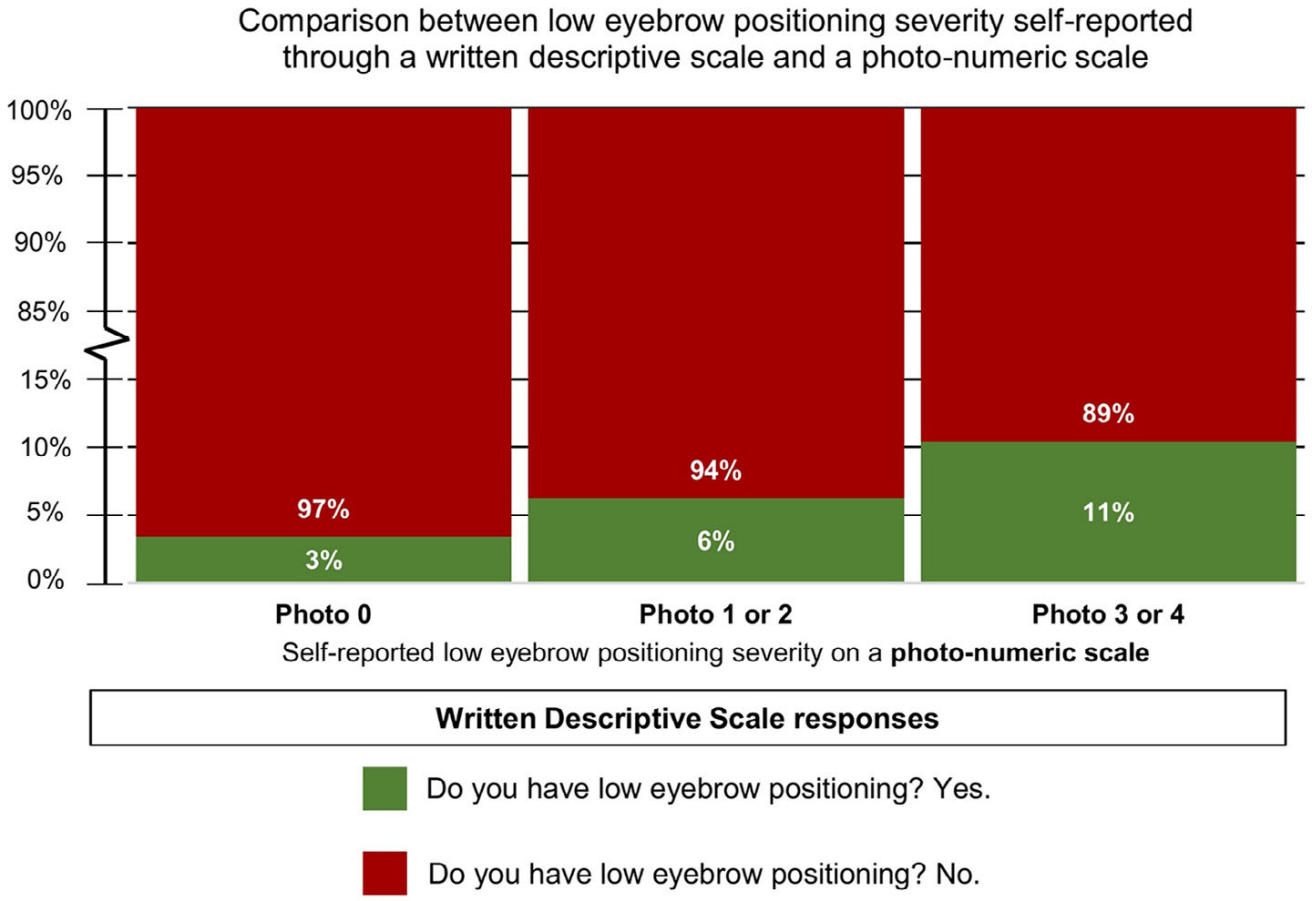
Comparison between self‐reported eyebrow positioning on a written descriptive scale (i.e., Do you have low eyebrow positioning?) and self‐reported eyebrow positioning on a photo‐numeric scale. The photo‐numeric scale for eyebrow positioning consists of five levels ranging from Photo 0 (high eyebrow positioning) to Photo 4 (low eyebrow positioning).

The majority (97%) of participants who identified with high eyebrow positioning (i.e., photo 0) also responded with a ‘no’ to ‘Do you have low eyebrow positioning?’ on the written descriptive scale (Figure 1). Among participants who identified with mid and low eyebrow positioning (i.e., photo 1 and above), 7% of them also responded with a ‘yes’ to the written descriptive scale question (Figure 1). Eyebrow positioning can be further divided into three levels: high eyebrow positioning (i.e., photo 0), mid eyebrow positioning (i.e., photos 1 and 2), and low eyebrow positioning (i.e., photos 3 and 4). About 6% of participants who identified with mid eyebrow positioning (i.e., photos 1 and 2) also responded with a ‘yes’ to the question ‘Do you have low eyebrow positioning?’ (Figure 4). About 11% of participants who identified with low eyebrow positioning (i.e., photos 3 and 4) also responded with a ‘yes’ to the same question (Figure 4).

## 4 DISCUSSION

### 4.1 Participant demographics

Participants of diverse ethnicities are recruited from Singapore and Malaysia. This is possible as both Singapore and Malaysia are racially diverse countries. However, an unequal proportion and sampling probability exists among some races. The ethnic Chinese group is the only major racial group with a large enough sample size. We therefore focused only on the ethnic Chinese. We obtained skin ageing data from 2885 participants to analyse in our current study. While we only focused on the ethnic Chinese in this study, we intend to conduct future analyses and description of skin ageing in other races (e.g., Malays and Indians) when the number of participants of the other races grows sufficiently large through progressive, annual recruitment drives to expand the Singapore/Malaysia Cross‐sectional Genetics Epidemiology Study (SMCGES) cohort.

### 4.2 Studying phenotypes through written descriptive scales and photo‐numeric scales

In our recent systematic review of skin ageing,^10^ we found that the quantification of skin ageing phenotypes in the field can be broadly classified into two categories: quantification using a written descriptive scale and quantification using a photo‐numeric scale.

Written descriptive measurements can take the form of dichotomous questions (e.g., ‘Have you ever been diagnosed with basal cell carcinoma?^2^) or multiple‐choice questions with increasing levels of severity (e.g., Does your face often become red at times (temperature differences, tension) other than alcohol consumption? Three choices are provided: 1. The face does not become red, 2. It sometimes becomes red, and 3. It tends to become red.^3^). In our study, we constructed dichotomous questions for our written descriptive scale. This is because by having just two choices (yes or no) for each phenotype, we aim to reduce the mental load involved and enable participants to make a clear and decisive choice.

When skin ageing phenotypes are quantified using a photo‐numeric scale, participants are shown a set of photographs in which successive photographs show increased extents of a particular form of skin ageing (e.g., increasingly severe eyebags). Analysing skin ageing phenotypes through self‐reported data on photo‐numeric scales is an established technique that has previously been done for pigment spots,^3^ freckles,^11^ and skin colour.^12^

### 4.3 Studying skin sagging phenotypes

We previously discussed in detail two forms of skin ageing: wrinkles^4^ and photo‐ageing.^5^ In this study, we will discuss yet another form of skin ageing: sagging of the skin. Skin sagging will be discussed from the perspective of three sagging phenotypes around the eyes. They are eyebags, droopy eyelids, and low eyebrow positioning. We focused on these three specific phenotypes because they are relatively simple to understand and describe in words. Since written descriptive measurements depend heavily on using words to convey the intent of the questions, we hypothesise that phenotypes which are easy to describe in words perform better on a written descriptive scale as compared to phenotypes which are difficult to describe in words.

### 4.4 Studying self‐reported phenotypes

Using phenotypes graded by trained assessors for comparison, we have previously found that self‐reported phenotypes provide a satisfactory approximation of the phenotype scores given by our trained assessors.^6^ Here, we will briefly summarise the relevant findings for skin sagging from our earlier study. We have previously reported good concordance between self‐reported data and evaluations by our trained assessors for skin ageing phenotypes around the eyes. We found a moderate concordance for eyebags (Spearman's Rank Correlation = 0.31–0.43, *p*‐value < 0.001), droopy eyelids (Spearman's rank correlation = 0.26–0.34, *p*‐value < 0.001), and eyebrow positioning (Spearman's rank correlation = 0.22–0.34, *p*‐value < 0.001). We interpret these findings to indicate that skin sagging phenotypes around the eyes can be satisfactorily approximated by self‐reported photo‐numeric scale data.

We explored the feasibility of combining photo‐numeric scales with descriptive scales for evaluating eyebags (Table S2a–c), droopy eyelids (Table S3a–c), and low eyebrow positioning (Table S4a–c). We assessed that these combinatorial scales have limited success. We found that a combination of the written descriptive scale and photo‐numeric scale attains a maximum correlation of 0.38 (*p*‐value < 0.001) for eyebags, 0.30 (*p*‐value < 0.001) for droopy eyelids, and 0.30 (*p*‐value < 0.001) for eyebrow positioning.

The main weakness of our validation study^6^ is that while the validation study uses a large sample size of 1081 ethnic Chinese participants, scaling this further up to our full dataset of 2885 ethnic Chinese participants is labour‐intensive and consequentially, challenging to deliver any findings in a timely manner. Meanwhile, we also published a meta‐analysis earlier^13^ that found significant epidemiological risk factors (e.g., age, gender, ethnicity, air pollution, smoking, nutrition, and Sun exposure) which influence the development of skin ageing phenotypes. Using self‐reported skin ageing data enables us to scale up our findings in large‐scale epidemiological studies, such as in a population of 2885 participants, in a reasonable time frame. This in turn enables us to explore in a larger population how risk factors are associated with different forms of skin ageing.

### 4.5 Skin sagging phenotypes

Our main objective in this study is to compare the performance of written descriptive scales and photo‐numeric scales. As the written descriptive scales only presents two choices (i.e., presence or absence of a skin sagging phenotype), this significantly reduces the mental load and should enable participants to make a decisive choice. Due to this decisiveness, we hypothesise that most participants who choose ‘no’ on the written descriptive scale will also choose the photo which shows no skin sagging. Similarly, most participants who choose ‘yes’ on the written descriptive scale will also choose one of the photos which shows skin sagging.

#### 4.5.1 Eyebags

Our hypothesis is correct for eyebags: eyebags evaluated using a photo‐numeric scale are moderately correlated with eyebags evaluated using a written descriptive scale (Spearman's rank correlation = 0.25, *p*‐value < 0.001). We found that a majority (61%) of participants who choose ‘no’ to the question ‘Do you have eyebags?’ also choose the photo with no eyebags (i.e., photo 0) (Figure 1). Likewise, we found that a majority (60%) of participants who choose ‘yes’ to the question ‘Do you have eyebags?’ also choose photos which show some degree of eyebags (i.e., photos 1–6) (Figure 1). Our results indicate that the term, ‘eyebags’, can successfully convey to the participants the concept of skin below the eyes sagging downwards.

Here, we further study participants with mild, moderate, and severe eyebags. There is a clearly increasing and persisting trend between choosing ‘yes’ on the written descriptive scale and choosing the photos which show mild, moderate, and severe eyebags. On a photo‐numeric scale from 0 to 6 in which photo 0 shows no eyebags and photo 6 shows severe eyebags, the percentage of respondents who choose ‘yes’ to the written descriptive scale question on whether they have eyebags increases progressively from photo 0 (39%) to photo 1 (50%), photo 2 (68%), photo 3 (74%), photo 4 (75%), photo 5 (81%), and photo 6 (92%) (Figure 2). This means that almost all (92%) participants who self‐report that the eyebags in photo 6 best represent their skin also choose ‘yes’ when asked ‘Do you have eyebags?’ (Figure 2).

#### 4.5.2 Droopy eyelids

Droopy eyelids as measured by the written descriptive scale is correlated with measurements made by the photo‐numeric scale (Spearman's Rank Correlation = 0.05, *p*‐value < 0.05), but weakly. We found that while some participants (14%) self‐report ‘yes’ to having droopy eyelids despite choosing photo 0 (i.e., the photo which shows no droopy eyelids), the proportion of participants that self‐reported ‘yes’ to having droopy eyelids and also choose a droopy eyelid photo (i.e., photos 1 and above) is higher (21%) (Figure 1). Our results indicate that the term, ‘droopy eyelids’, can adequately convey to the participants the concept of eyelids which sag downwards.

Next, we split participants into two groups. One group identified with photo 1 (mild droopy eyelids) and the other group identified with photo 2 (severe droopy eyelids). We found that participants who identified with photo 1 are more likely (21%) to choose ‘yes’ when asked ‘Do you have droopy eyelids’ (Figure 3). Our results indicate that when presented with two choices (i.e., ‘Do you have droopy eyelids? Yes or no), choosing ‘yes’ corresponds the most strongly to photo 1, not photo 2, on the photo‐numeric scale. This is important because one of the design objectives of the droopy eyelids photo‐numeric scale is to split participants into two groups: no droopy eyelids (i.e., photo 0) and some droopy eyelids (i.e., anything other than photo 0). We have shown that this exact wording of the question ‘Do you have droopy eyelids?’ is effective in drawing a distinction between participants who choose photo 0 and participants who choose photo 1 and above.

#### 4.5.3 Eyebrow positioning

Eyebrow positioning as measured by the written descriptive scale is correlated with measurements made by the photo‐numeric scale (Spearman's Rank Correlation = 0.08, *p*‐value < 0.001), but weakly. Like for droopy eyelids, we found that while a very small proportion of participants (3%) self‐reported ‘yes’ to having a low eyebrow positioning despite choosing photo 0 (i.e., the photo which shows the highest eyebrow positioning), the proportion of participants that self‐reported ‘yes’ to having a low eyebrow positioning and also choose a low eyebrow positioning photo (i.e., photos 1 and above) is higher (7%) (Figure 1). Our results indicate that the term, ‘low eyebrow positioning’, can adequately convey to the participants the concept of eyebrows sagging close to the eyes.

Next, we split participants into five groups (i.e., photos 0–4) based on how close their eyebrows sag relative to their eyes. The results for photos 1 and 2 are similar and will be analysed together. Likewise, the results for photos 3 and 4 are also analysed together. We now have three groups: group 1 with high eyebrow positioning (photo 0), group 2 with mid eyebrow positioning (photos 1 or 2), and group 3 with low eyebrow positioning (photos 3 or 4). We found that the percentage of respondents who choose ‘yes’ to the question ‘Do you have low eyebrow positioning?’ increases progressively from group 1 (3%) to group 2 (6%) to group 3 (11%) (Figure 4). Our results indicate that this exact wording of the question ‘Do you have low eyebrow positioning?’ triggers participants into categorising their own eyebrow positioning into one of three groups: high, mid, or low eyebrow positioning. Taking the high eyebrow positioning group as our reference, participants who categorised themselves as mid eyebrow positioning are more likely (6%) to respond with ‘yes’ to the question ‘Do you have low eyebrow positioning?’. Participants who categorised themselves as low eyebrow positioning are even more likely (11%) to respond with ‘yes’ to the same question (Figure 4).

### 4.6 Strengths and limitations of using written descriptive scales

In this study, we have discussed at length how written descriptive scale questions can deliver results with the same trends as photo‐numeric scale questions. The exact wording of some questions (e.g., ‘Do you have droopy eyelids?’) can distinguish between the presence and absence of a phenotype in the same way that a photo‐numeric scale question does. The exact wording of some other questions (e.g., ‘Do you have eyebags?’ and ‘Do you have low eyebrow positioning?’) generates a stepwise increasing trend which resembles the property of photo‐numeric scales. Furthermore, written descriptive scale questions are easier to deploy and replicate in questionnaires because they are short and concise. These may be some of the reasons justifying the continued usage of written descriptive scale questions by the field to assess skin ageing phenotypes.^1^, ^2^, ^3^, ^11^, ^12^, ^14^, ^15^

However, the key drawback of using written descriptive scale questions lies in how its very design makes it heavily reliant on descriptive words. Even though we hypothesised earlier that phenotypes which are easy to describe in words perform better on a written descriptive scale as compared to phenotypes which are difficult to describe in words, we observed a noticeable variation in how the three skin sagging phenotypes performed. We originally believed that the terms eyebags, droopy eyelids, and low eyebrow positioning are simple and well‐understood and can therefore evoke the same imagery as their respective photo‐numeric scales. We found that this is true only for eyebags (Spearman's Rank Correlation = 0.25, *p*‐value < 0.001) (Table 2). The correlations for droopy eyelids (Spearman's Rank Correlation = 0.05, *p*‐value < 0.05) and low eyebrow positioning (Spearman's Rank Correlation = 0.08, *p*‐value < 0.001) are weak (Table 2) and this might be because the terms droopy eyelids and low eyebrow positioning generate a different mental imagery from what is visually presented in the photo‐numeric scales. As a result, the written descriptive scale responses and the photo‐numeric scale responses are not strongly correlated. Only in the minority of participants where both imagery overlap do we see interesting results such as stepwise increasing trends which we have earlier discussed. This shortcoming of written descriptive scales makes it a challenge to use written descriptive scales to identify other skin ageing phenotypes, some of which are not that easy to describe in words. These may be some of the reasons motivating the field to assess skin ageing phenotypes using other methods. One such alternative method, photo‐numeric scales, circumvents words completely by using photographs instead. However, using photo‐numeric scales has its own set of challenges, such as accounting for gender and ethnic variations in the development and progression of skin ageing phenotypes, both of which have been discussed in detail in our earlier studies.^4^, ^5^

### 4.7 Effects of using anti‐ageing skincare creams, substances, or therapies on skin sagging

We then wondered about the effect of anti‐ageing interventions across age groups. We found that anti‐ageing interventions have beneficial effects for eyebags, droopy eyelids, and eyebrow positioning.

Firstly, we found that among participants who use anti‐ageing interventions, eyebags do not significantly increase in severity from 18 to 45 years old (Chi‐square test *p*‐value ≥0.10) (Figure S4a). In contrast, among participants who do not use anti‐ageing interventions, eyebags steadily increase in severity from 18 to 45 years old (Chi‐square test *p*‐value ≤ 0.05) (Figure S5a).

Secondly, we found that among participants who use anti‐ageing interventions, droopy eyelids do not significantly increase in severity from 31 to 45 years old (Chi‐square test *p*‐value ≥0.06) (Figure S4b). In contrast, among participants who do not use anti‐ageing interventions, droopy eyelids steadily increase in severity from 31 to 45 years old (Chi‐square test *p*‐value < 0.001) (Figure S5b).

Lastly, we found that while eyebrow positioning does not significantly increase in severity from 21 to 35 years old for non‐users of anti‐ageing interventions (Chi‐square test p‐value≥0.07) (Figure S5c), this youthfulness persists longer from 21 to 40 years old for users of anti‐ageing interventions (Chi‐square test p‐value≥0.08) (Figure S4c).

Analysing these results as a whole, anti‐ageing interventions delay the onset or severity of skin sagging phenotypes around the eyes. However, as eyebags, droopy eyelids, and lower eyebrow positioning have individual developmental timelines, the beneficial effects of anti‐ageing interventions on these three phenotypes become the most apparent at different age ranges. Anti‐ageing interventions are associated with delays in the onset and progression of eyebags from 18 to 45 years old, associated with delays in the onset and progression of droopy eyelids from 31 to 45 years old, and associated with an extended delay in the progression of low eyebrow positioning by five years from 21 to 35 years old to 21 to 40 years old.

Our results show that the appropriate age range for studying some skin ageing phenotypes, such as eyebags, and the effect of anti‐ageing interventions on these phenotypes, can be as young as 18 to 45 years old. A potential limitation for our data is that the data collected does not distinguish between anti‐ageing interventions around the eye region and anti‐ageing interventions elsewhere on the skin. However, we have instead shown that anti‐ageing interventions in general have significant benefits to skin sagging phenotypes around the eyes.

### 4.8 Effects of digital screen time on skin sagging

The duration of digital screen time usage is a potential contributing factor to the development of skin sagging phenotypes around the eyes. We found that after stratifying for the duration of digital screen time usage, the proportion of participants with lower eyebrow positioning is significantly different between participants with 1 h or less per day of digital screen time and participants with 1–3 h of digital screen time per day (Chi‐square test *p*‐value = 0.002) (Figure S6c). The duration of digital screen time spent per day is not significantly associated with eyebags (Figure S6a) and droopy eyelids (Figure S6b).

We also analysed digital screen time in detail. 21–25‐year‐old participants who spend less than 1 h in front of the television or computer every day have significantly lower eyebrow positioning (i.e., more sagging) than 18–20‐year‐old participants (Chi‐square test *p*‐value = 0.02). Likewise, 26–30‐year‐old participants who spend less than 1 h in front of the television or computer every day also have significantly lower eyebrow positioning than 21–25‐year‐old participants (Chi‐square test *p*‐value = 0.01) (Figure S7c). The trend is similar for 1–3 h of digital screen time. 21–25‐year‐old participants who spend 1–3 h in front of the television or computer every day have significantly lower eyebrow positioning than 18–20‐year‐old participants (Chi‐square test *p*‐value = 0.02). 26–30‐year‐old participants who spend 1–3 h in front of the television or computer every day also have significantly lower eyebrow positioning than 21–25‐year‐old participants (Chi‐square test *p*‐value = 0.04) (Figure S7c). Finally, 26–30‐year‐old participants who spend more than 5 h in front of the television or computer every day also have significantly lower eyebrow positioning than 21–25‐year‐old participants (Chi‐square test *p*‐value = 0.01) (Figure S7c).

The effect of digital screen time on eyebags is significantly different between 18–20‐year‐old participants who spend 1–3 h in front of the television or computer every day and 21–25‐year‐old participants who spend 1–3 h in front of the television or computer every day (Chi‐square test *p*‐value = 0.04) (Figure S7a). The duration of digital screen time spent per day is not significantly associated with droopy eyelids (Figure S7b) even after stratifying by age groups.

Analysing our results as a whole, we show that a young cohort, comprising of participants aged 18–30 years old, is a suitable cohort for studying eyebrow positioning and the duration of digital screen time spent per day is significantly associated with this skin sagging phenotype.

## 5 CONCLUSION

In conclusion, we achieved various degrees of success in using written descriptive scales to quantify skin sagging phenotypes. We found that the term ‘eyebags’ is very well‐understood and a written descriptive scale for eyebags is moderately correlated with a photo‐numeric scale for eyebags. In contrast, the terms ‘droopy eyelids’ and ‘low eyebrow positioning’ may not be interpreted in the same way as what is shown on their respective photo‐numeric scales. While the written descriptive scale for ‘droopy eyelids’ and ‘low eyebrow positioning’ show some degree of success, these successes tend to be limited to a portion of the study subjects. Written descriptive scales for phenotypes which are difficult to describe in words will likely face greater challenges than the three sagging phenotypes that we have investigated here. This is because it may be difficult to use words to accurately convey the intended meaning. Using photo‐numeric scales to study skin ageing phenotypes is a promising solution which partially solves this problem. However, photo‐numeric scales bring new challenges such as gender and ethnic differences in skin ageing. Active development and validation of gender‐specific and ethnic‐specific photo‐numeric scales holds promise in mitigating these challenges. In the meantime, validating and adapting existing photo‐numeric scales to use in different population contexts appears to strike the best compromise between phenotypic identification and usability of the data.

Anti‐ageing interventions are associated with delays in the onset and progression of eyebags from 18 to 45 years old, associated with delays in the onset and progression of droopy eyelids from 31 to 45 years old, and associated with an extended delay in the progression of low eyebrow positioning by five years from 21 to 35 years old to 21 to 40 years old. Since eyebags, droopy eyelids, and lower eyebrow positioning have individual developmental timelines, the beneficial effects of anti‐ageing interventions on these three phenotypes become the most apparent at different age ranges.

Digital screen time, measured by the duration of exposure to the television or computer every day is significantly associated with eyebrow positioning. 26‐ to 30‐year‐old participants who spend less than 1 h in front of the television or computer every day have significantly lower eyebrow positioning (i.e., more sagging) than 21‐ to 25‐year‐old participants. These 21‐ to 25‐year‐old participants, in turn, have significantly lower eyebrow positioning than 18–20‐year‐old participants. Similar trends are observed among participants who spend 1–3 h in front of the television or computer every day. All in all, the duration of digital screen time usage is associated with skin sagging phenotypes around the eyes, even in young participants aged 18 to 30 years old.

## CONFLICT OF INTEREST STATEMENT

F.T.C reports grants from the National University of Singapore, Singapore Ministry of Education Academic Research Fund, Singapore Immunology Network, National Medical Research Council (NMRC) (Singapore), Biomedical Research Council (BMRC) (Singapore), National Research Foundation (NRF) (Singapore), Singapore Food Agency (SFA), and the Agency for Science Technology and Research (A*STAR) (Singapore), during the conduct of the study; and consulting fees from Sime Darby Technology Centre; First Resources Ltd; Genting Plantation, Olam International, and Syngenta Crop Protection, outside the submitted work. The other authors declare no other competing interests.

## ETHICS STATEMENT

This study was conducted in accordance with the principles of the Declaration of Helsinki and Good Clinical Practices, and in compliance with local regulatory requirements. The cross‐sectional studies in Singapore were conducted on the National University of Singapore (NUS) campus annually between 2005 and 2023 with the approval of the Institutional Review Board (Reference Code: NUS‐07‐023, NUS‐09‐256, NUS‐10‐445, NUS‐13‐075, NUS‐14‐150, and NUS‐18‐036) and by the Helsinki declaration, of which, participants between 2011 and 2022 participated in the current skin ageing study (Reference Code: NUS‐2020‐495). The cross‐sectional studies in Malaysia were held in Universiti Tunku Abdul Rahman (UTAR) Campus and Sunway University. Ethical approval was granted from the Scientific and Ethical Review Committee of UTAR (Reference Code: U/SERC/03/2016) and the Sunway University Research Ethics Committee (Reference Code: SUREC 2019/029 and SUREC 2022/049). Before the data collection, all participants involved signed an informed consent form.

## Supporting information

Supporting Information

Supporting Information

Supporting Information

Supporting Information

Supporting Information

## Data Availability

All data used and included in this study are available from the corresponding author (F.T.C.).
