# Supplementary figures and images for: Comparisons between eyebags, droopy eyelids, and eyebrow positioning identified by photo‐numeric scales or identified by written descriptive scales: Insights from the Singapore/Malaysia cross‐sectional genetics epidemiology study (SMCGES) cohort
Source: Skin Res Technol. 2024 Feb 20;30(2):e13620. doi: 10.1111/srt.13620 (PMC10878178; doi:10.1111/srt.13620)

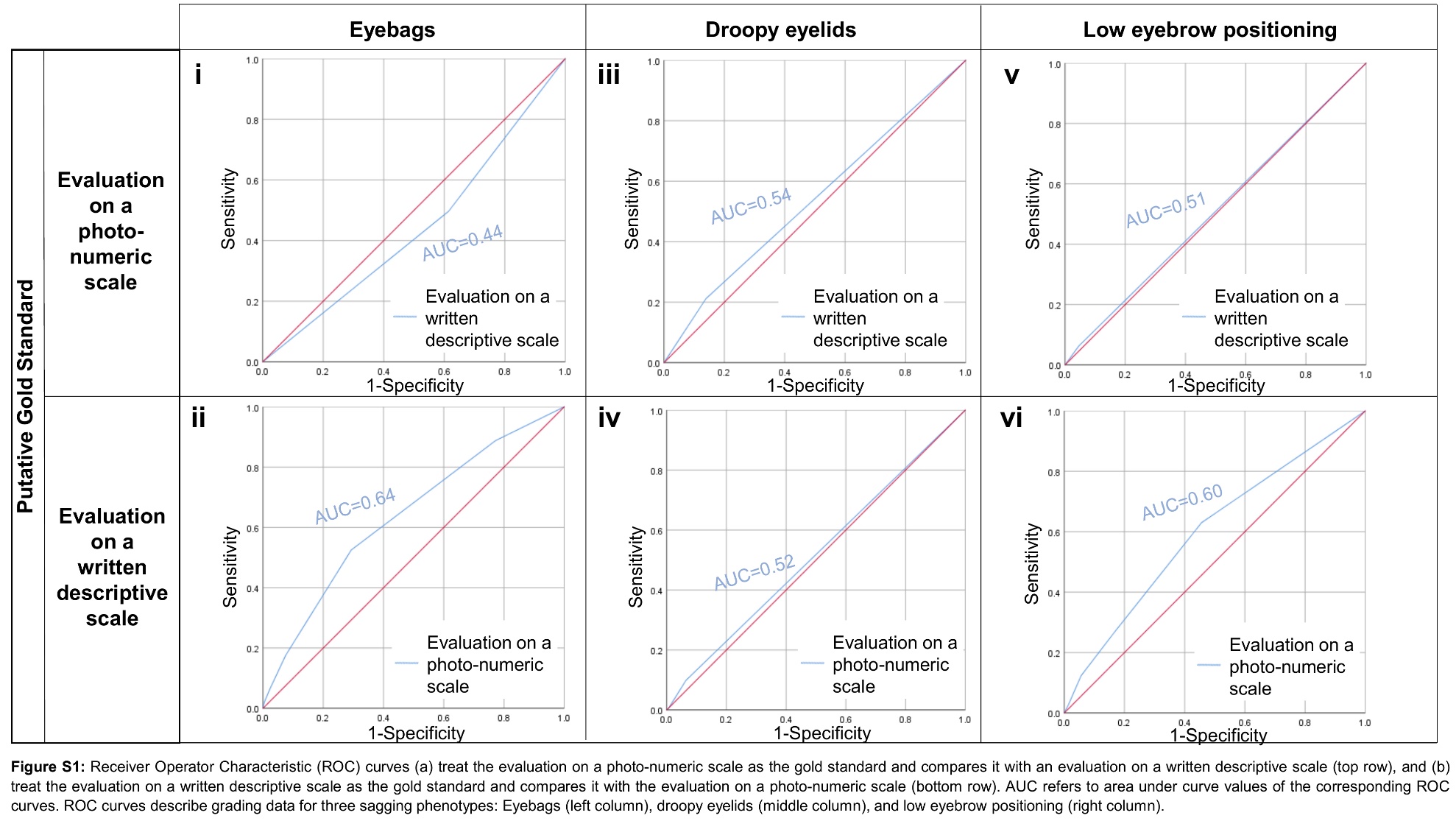


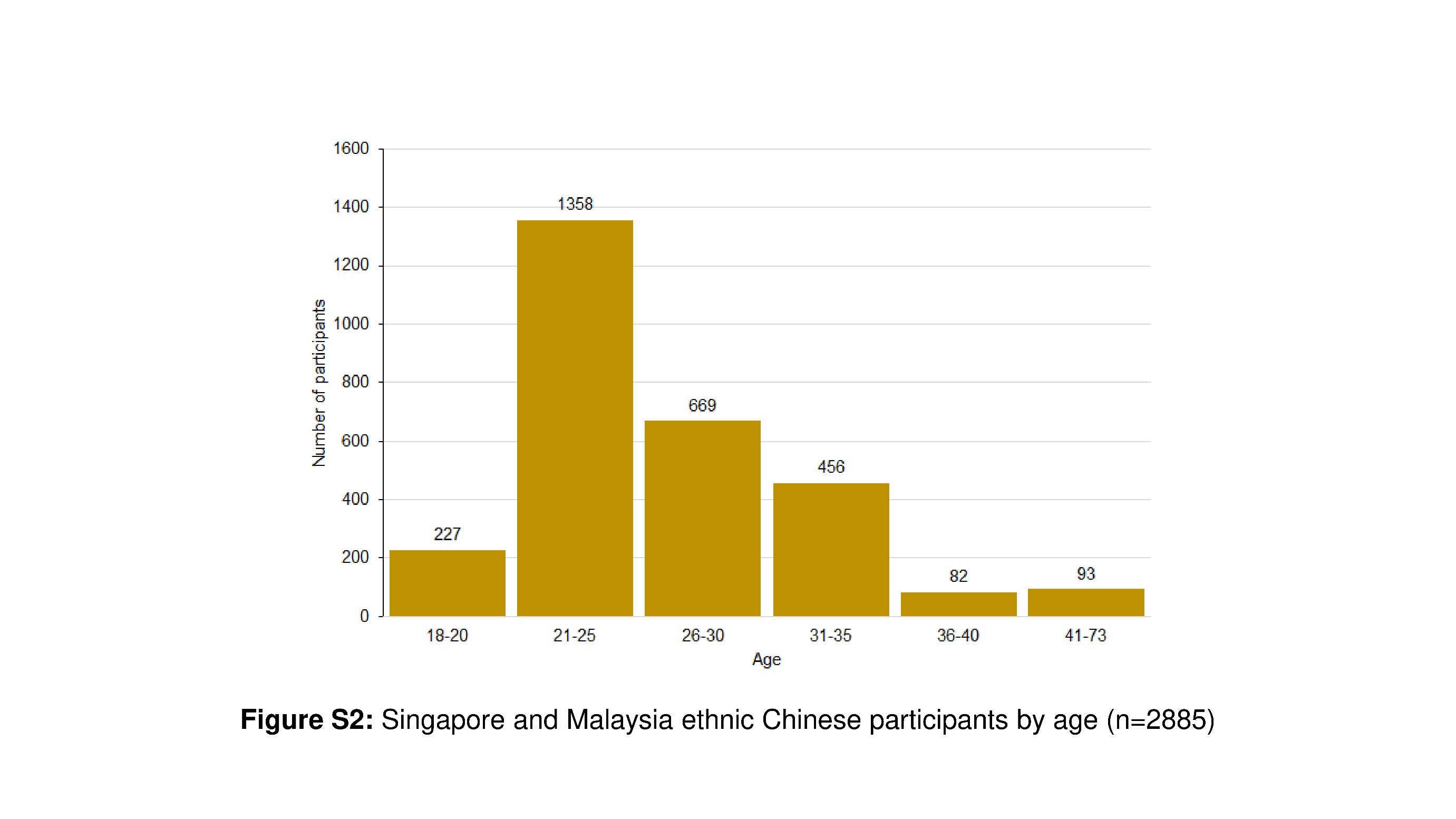


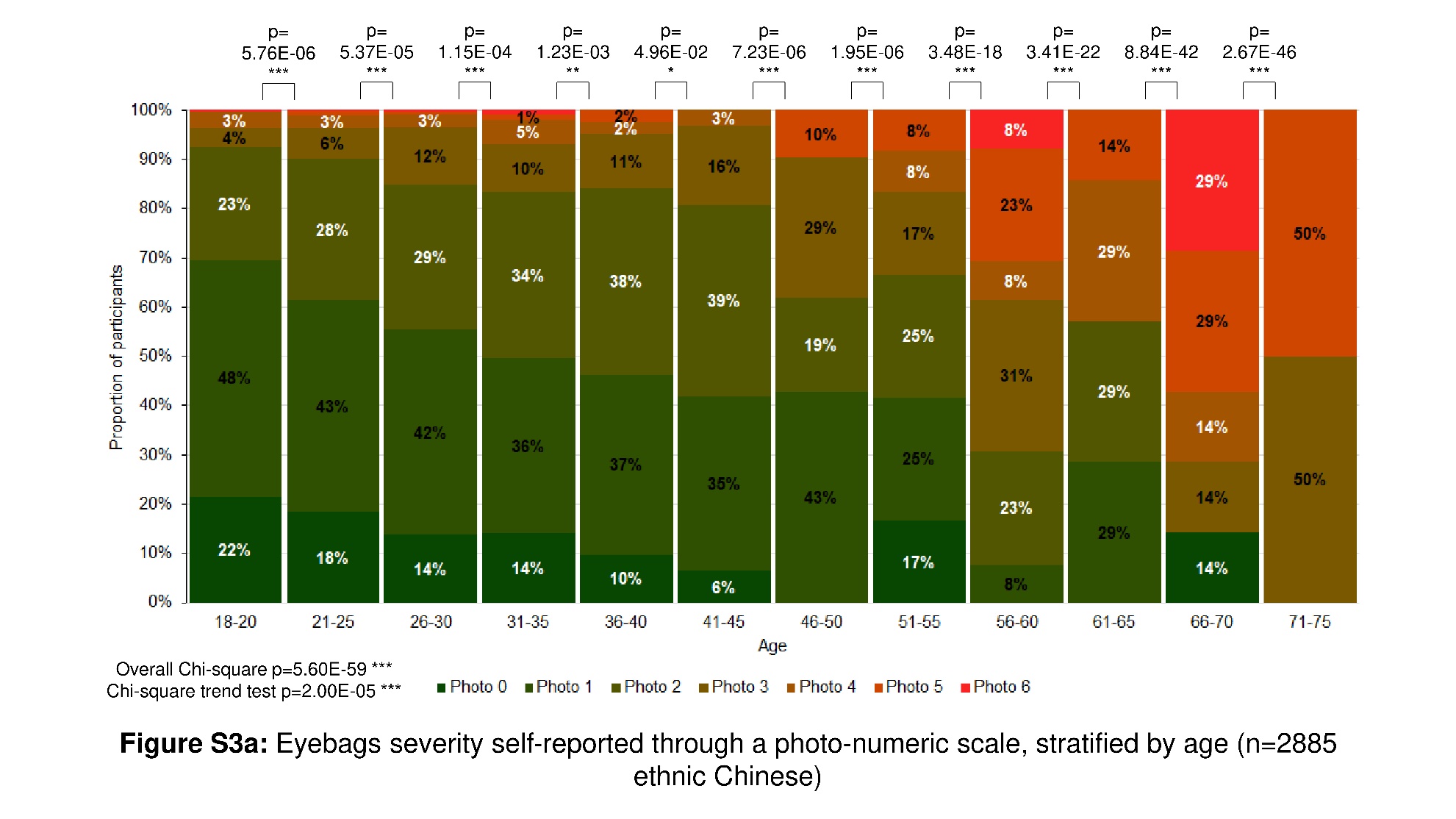


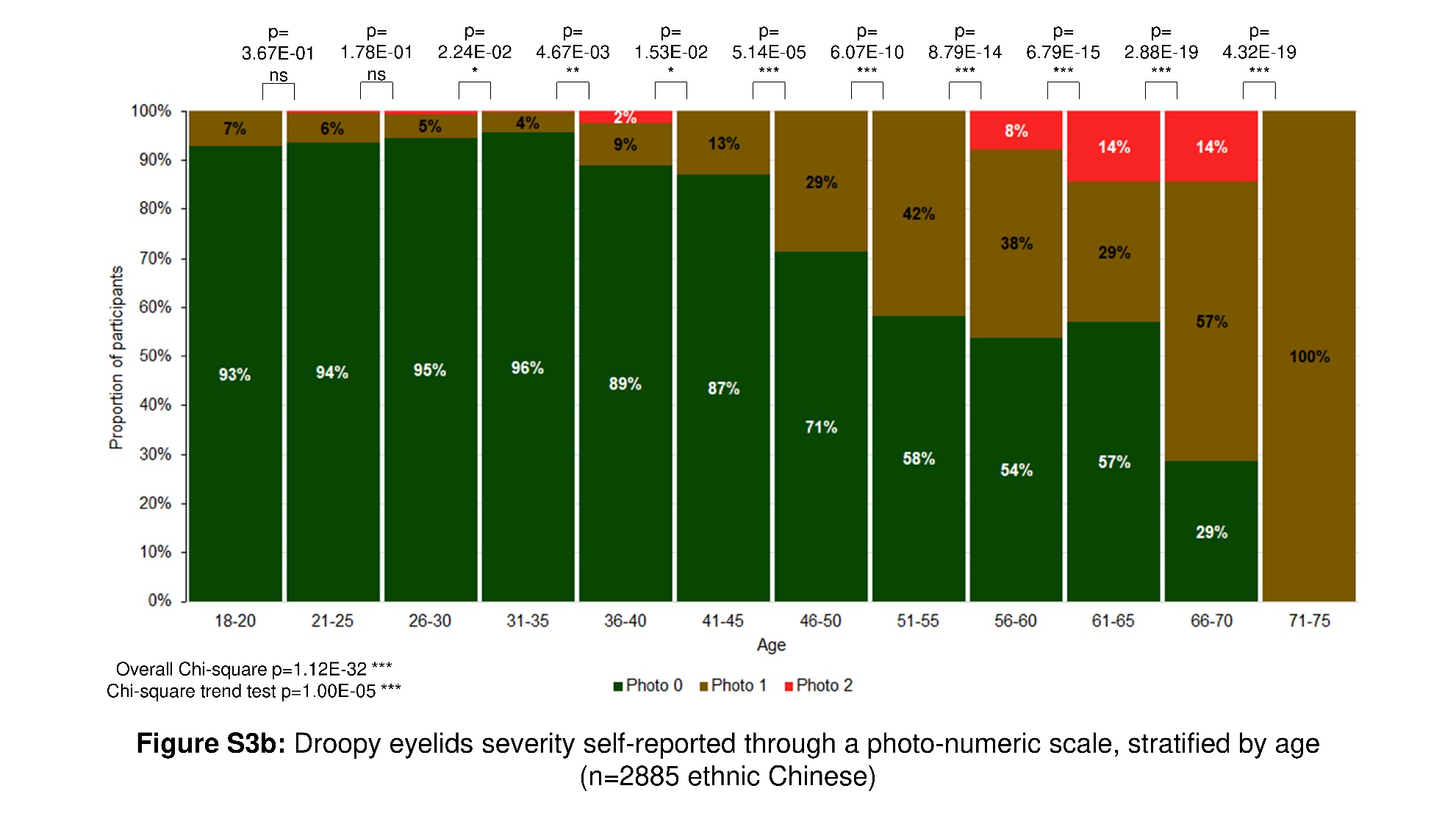


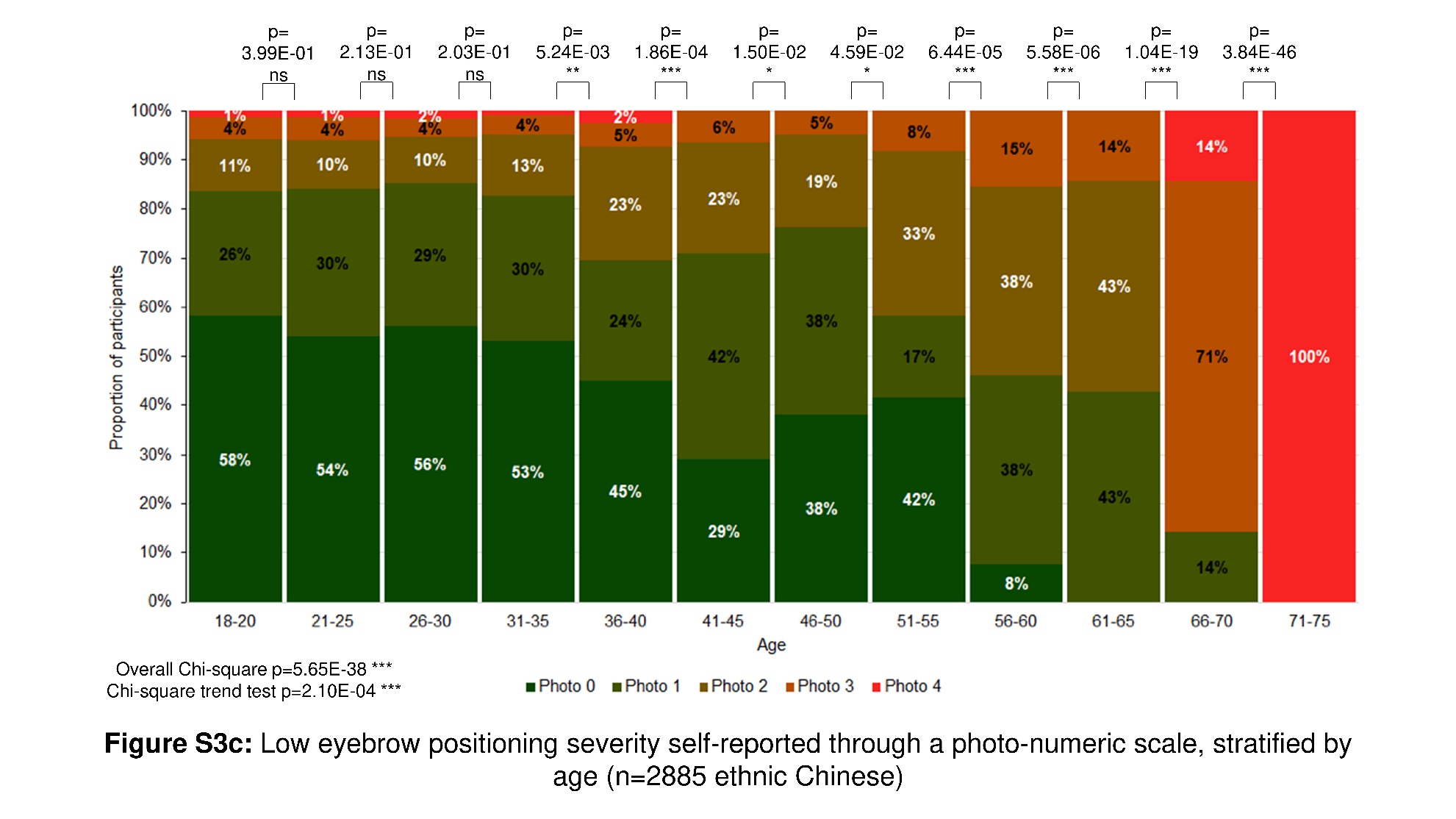


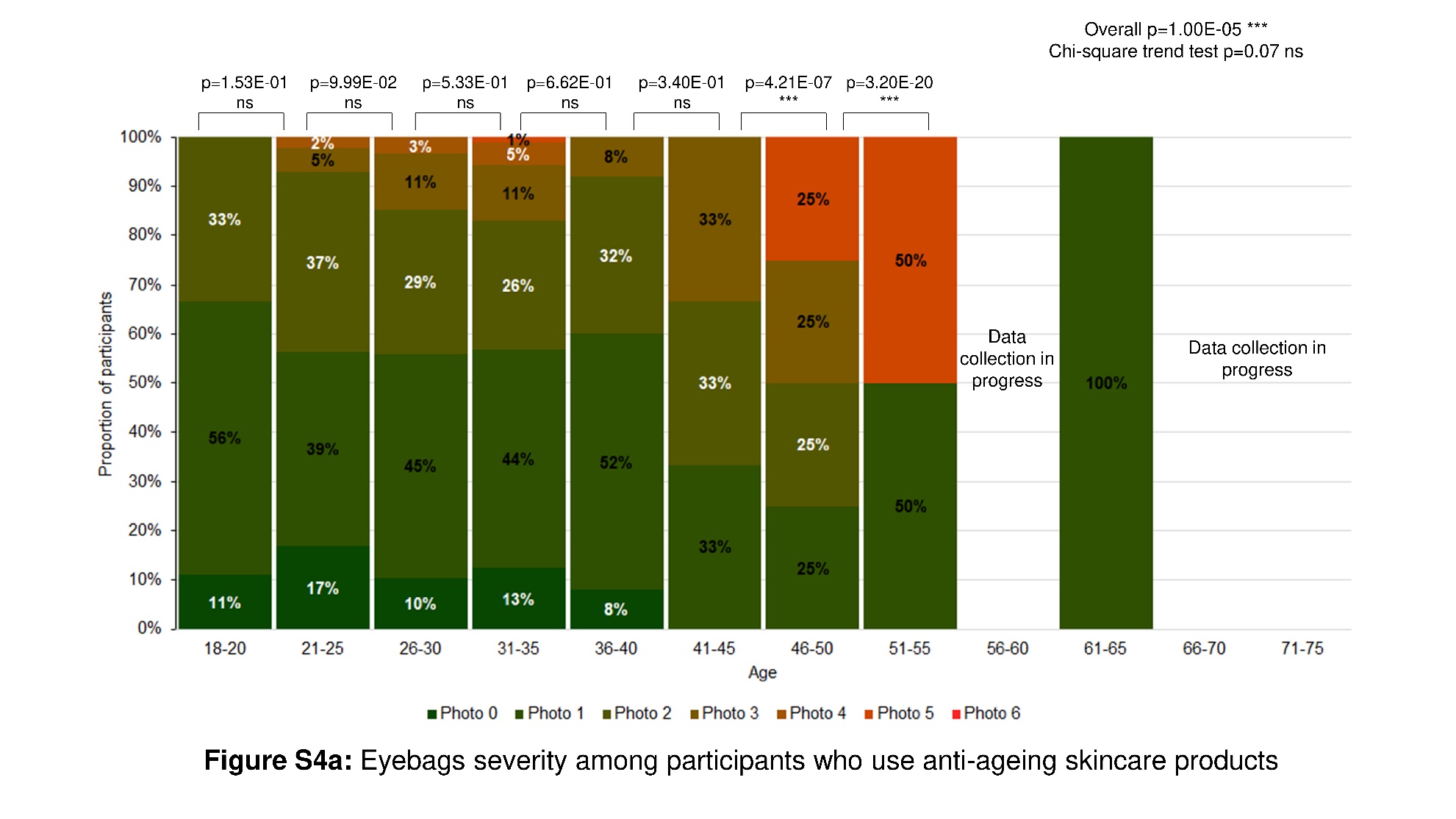


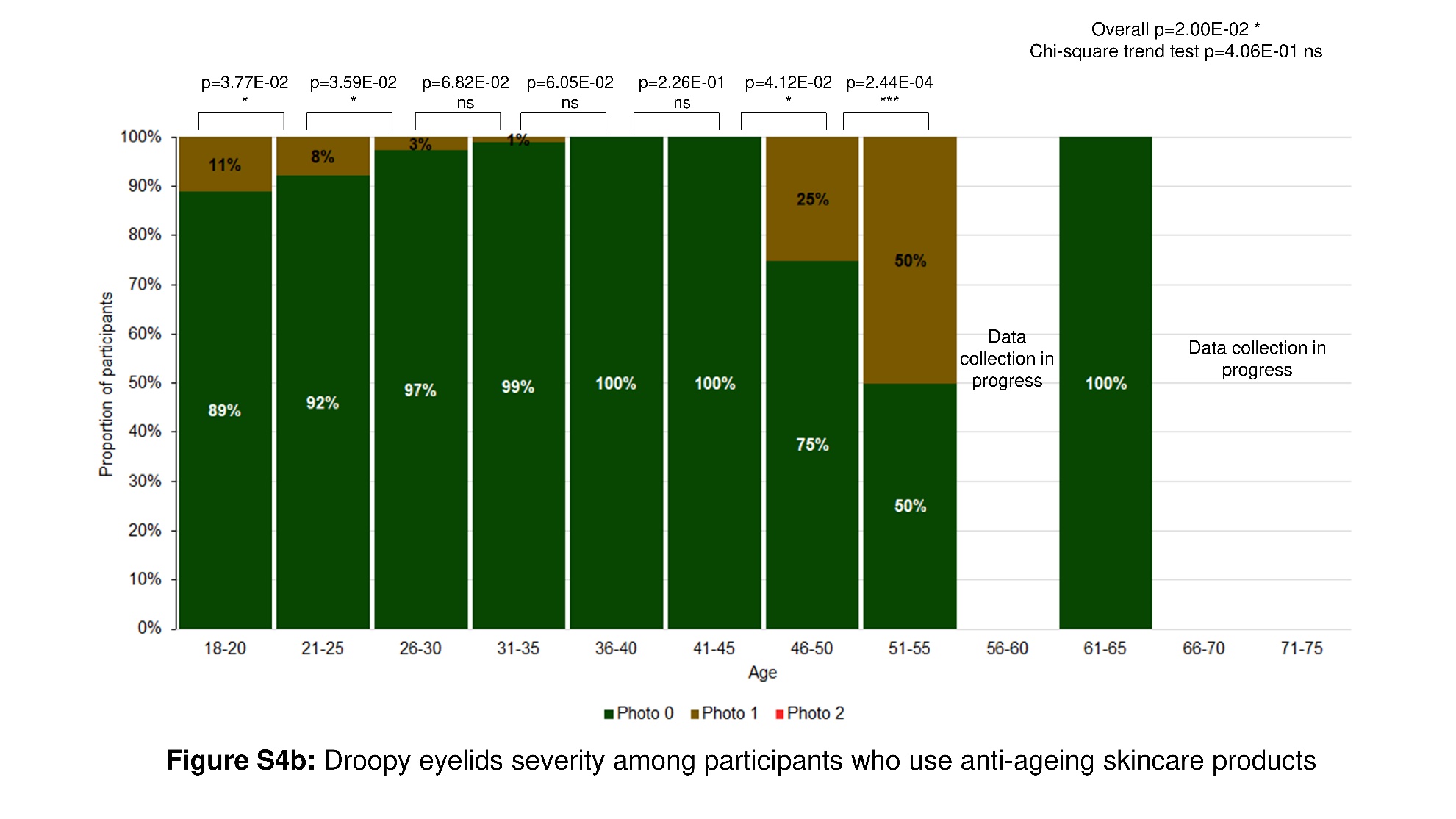


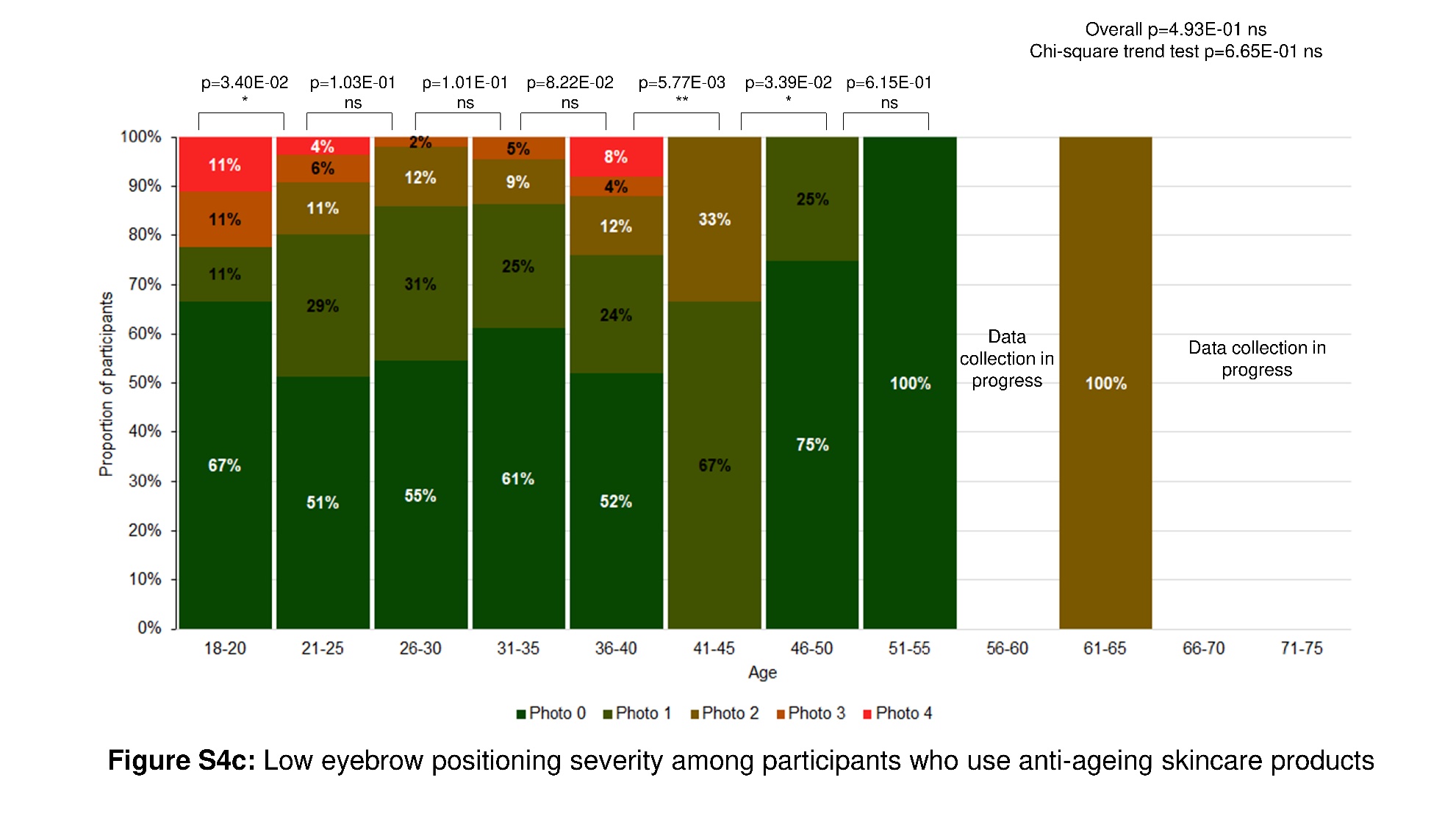


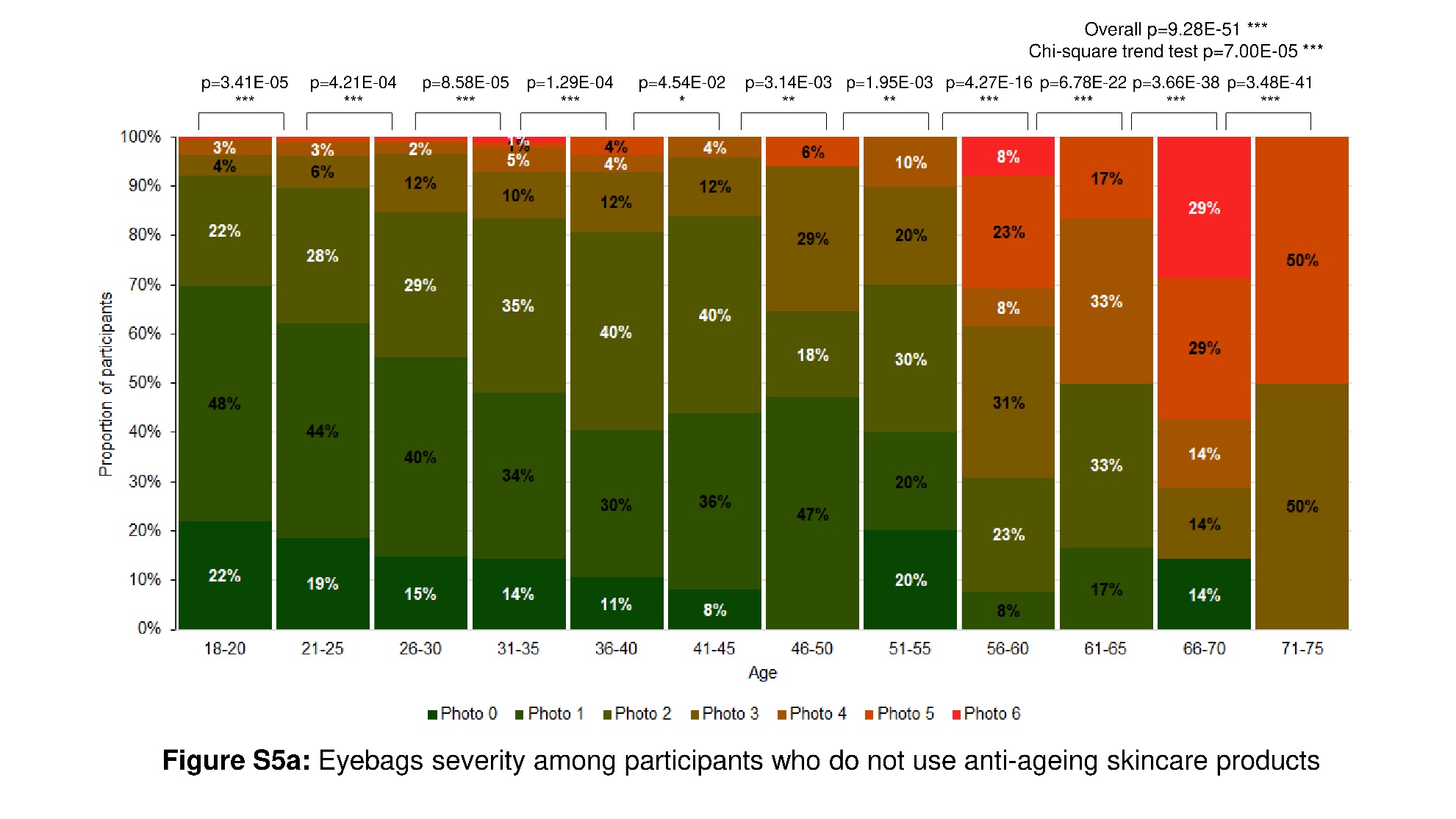


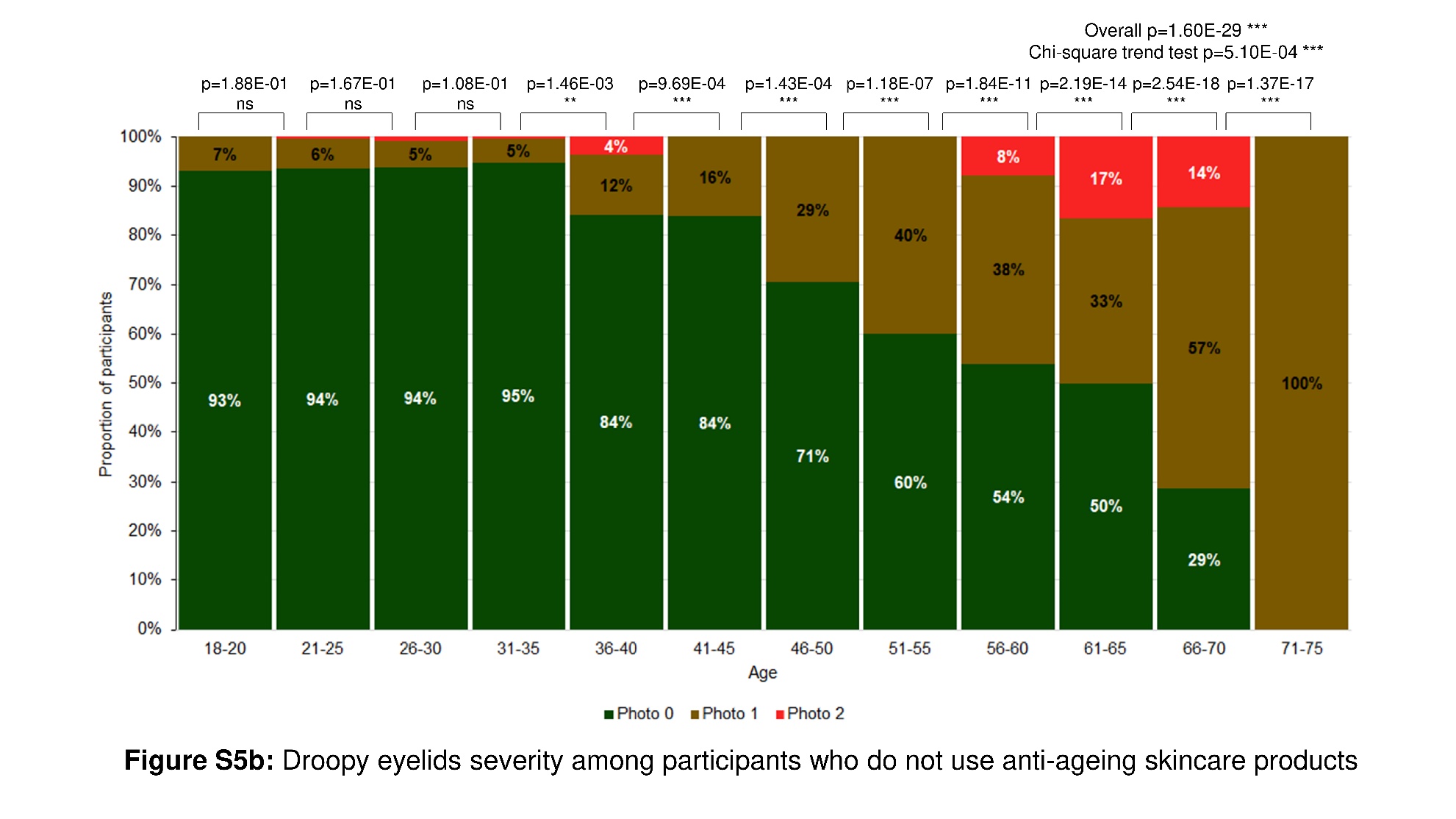


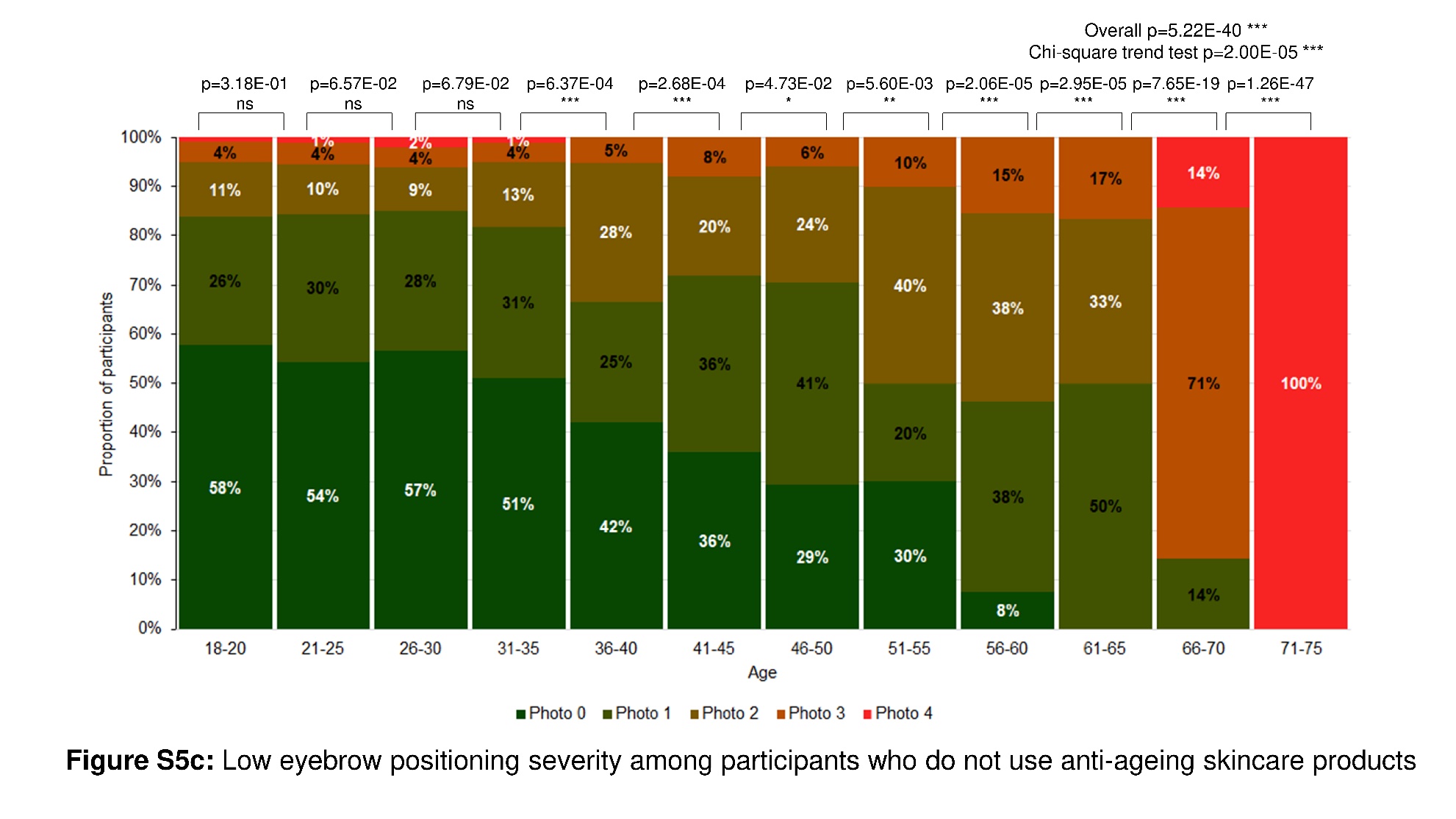


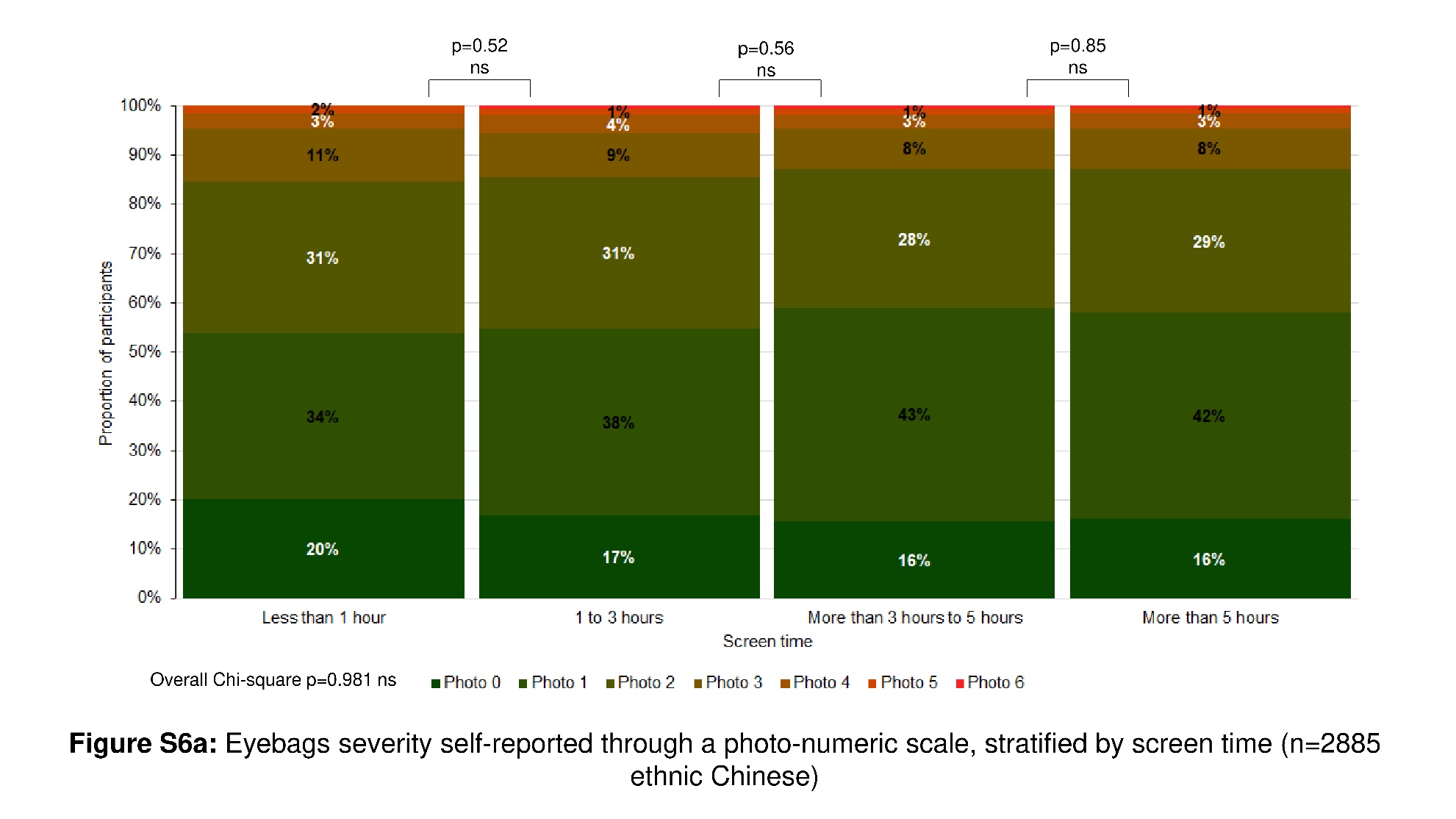


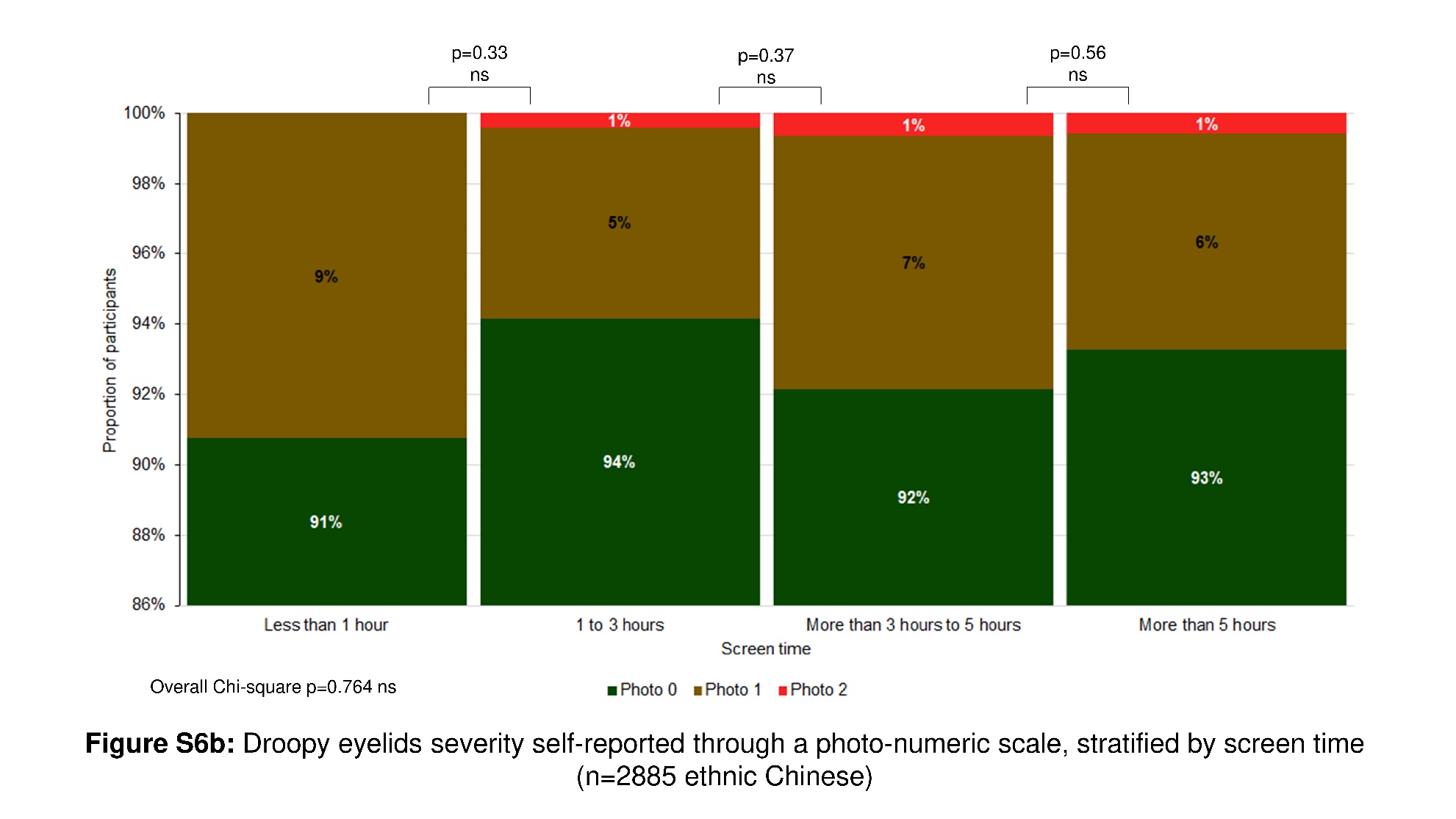


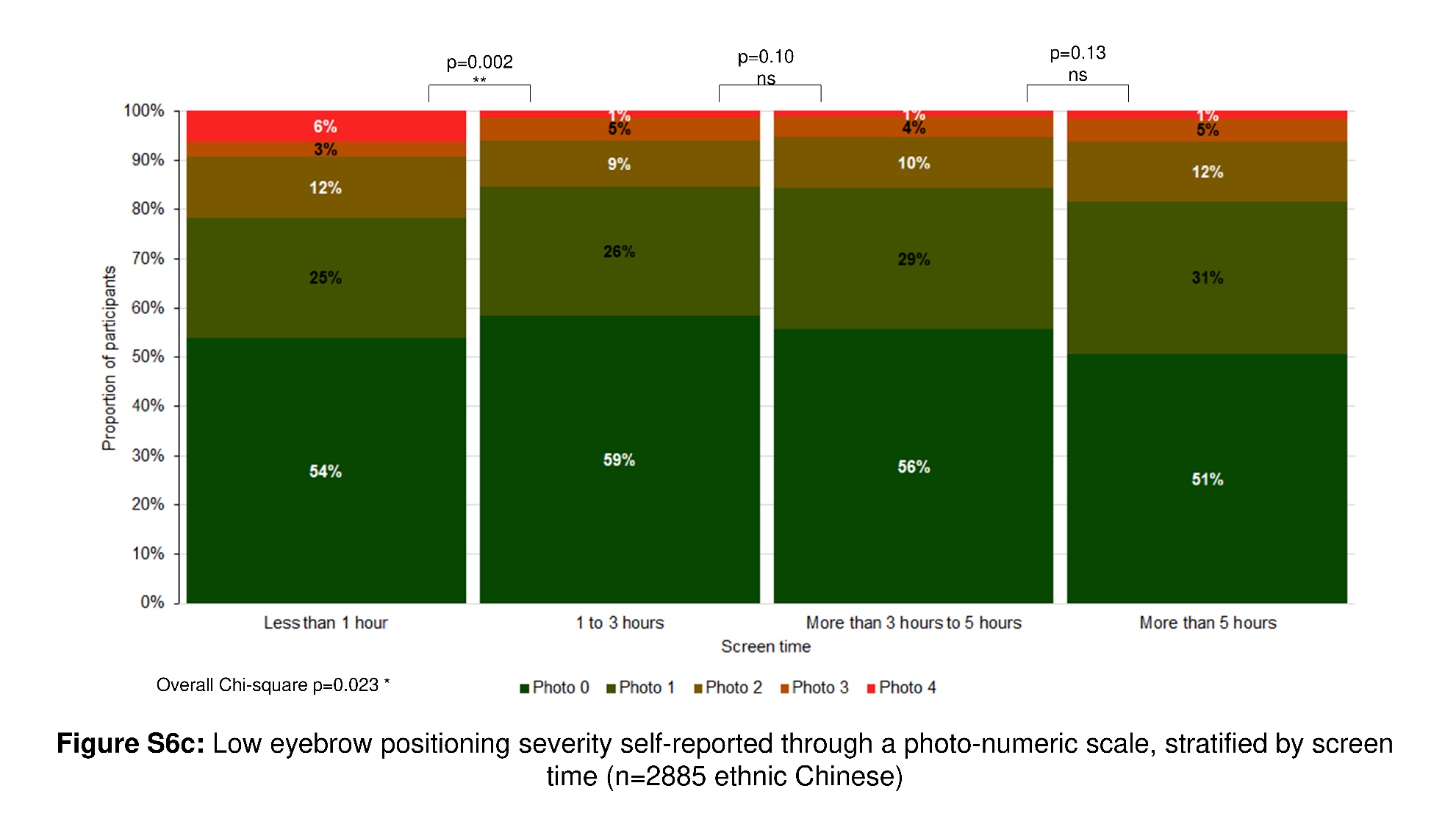


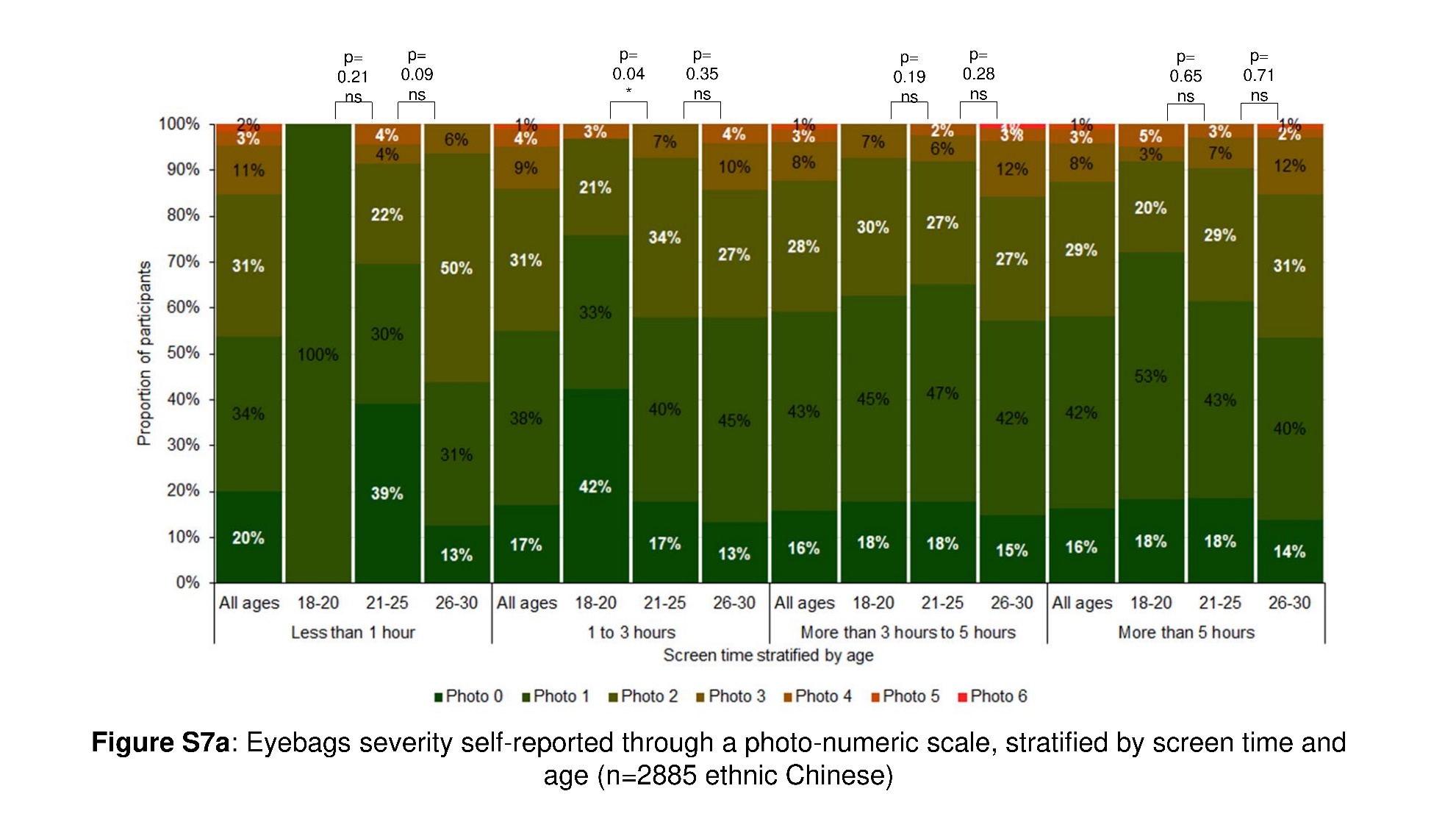


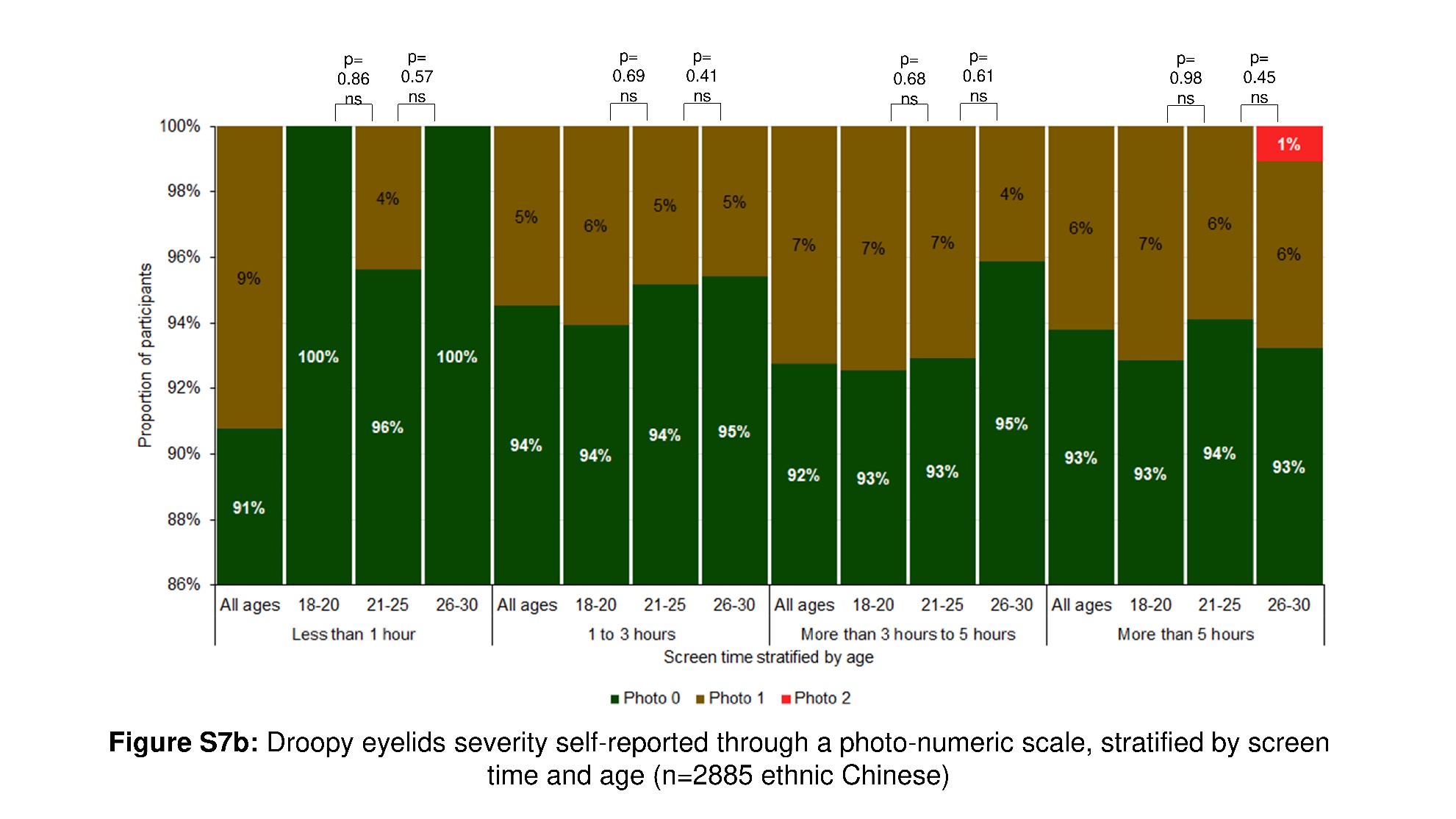


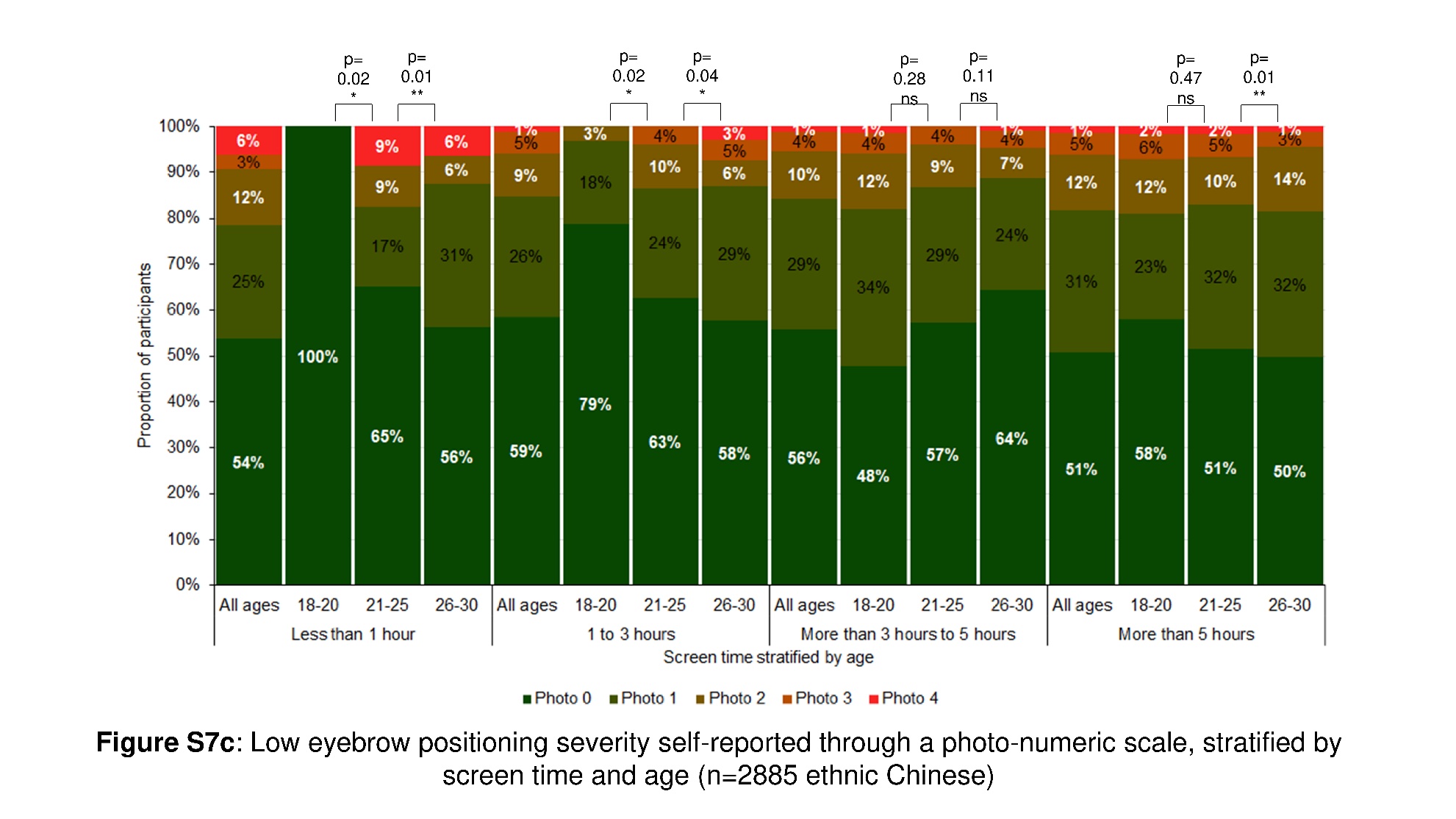

Supplement: Supplementary file 1 — Supporting Information [file SRT-30-e13620-s003.docx]
