# Supplementary material for: Comparisons between eyebags, droopy eyelids, and eyebrow positioning identified by photo‐numeric scales or identified by written descriptive scales: Insights from the Singapore/Malaysia cross‐sectional genetics epidemiology study (SMCGES) cohort
Source: Skin Res Technol. 2024 Feb 20;30(2):e13620. doi: 10.1111/srt.13620 (PMC10878178; doi:10.1111/srt.13620)
Supplement: Supplementary file 2 — Supporting Information [file SRT-30-e13620-s005.docx]

**Table S1:** Sources of the validated photo-numeric scales.

| **Phenotype** | **Title of the paper** | **Citation of the paper** |
| --- | --- | --- |
| Eyebags | Comparison of sagging at the cheek and lower eyelid between male and female faces. | Ezure, T., Yagi, E., Kunizawa, N., Hirao, T., and Amano, S. (2011). Comparison of sagging at the cheek and lower eyelid between male and female faces. *17*, 510–515. |
| Droopy eyelids | Cosmetic outcome of posterior approach ptosis surgery. | Goldberg, R.A., and Lew, H. (2011). Cosmetic outcome of posterior approach ptosis surgery. Trans. Am. Ophthalmol. Soc. *109*, 157–167. |
| Low eyebrow positioning | A Validated Brow Positioning Grading Scale | Carruthers, A., Carruthers, J., Hardas, B., Kaur, M., Goertelmeyer, R., Jones, D., Rzany, B., Cohen, J., Kerscher, M., Flynn, T.C., et al. (2008). A Validated Brow Positioning Grading Scale. *34*, S150–S154. |
