# Supplementary material for: Comparisons between eyebags, droopy eyelids, and eyebrow positioning identified by photo‐numeric scales or identified by written descriptive scales: Insights from the Singapore/Malaysia cross‐sectional genetics epidemiology study (SMCGES) cohort
Source: Skin Res Technol. 2024 Feb 20;30(2):e13620. doi: 10.1111/srt.13620 (PMC10878178; doi:10.1111/srt.13620)
Supplement: Supplementary file 3 — Supporting Information [file SRT-30-e13620-s004.docx]

**Table S2a**: Exploration of the feasibility of combining photo-numeric scales with descriptive scales (i.e., combinatorial scoring methods) for evaluating eyebags. Here, we explored combining the written and photo-numeric scale together and assessed this against the assessor-evaluated scoring.

| **Other Standard** | **Measure** | **Putative Gold Standard** | | | | | |
| --- | --- | --- | --- | --- | --- | --- | --- |
|  |  | Assessor-evaluated scoring via a photo-numeric scale (Lax definition) | | Assessor-evaluated scoring via a photo-numeric scale (Moderately-strict definition) | | Assessor-evaluated scoring via a photo-numeric scale (Strict definition) | |
| Self-reported scoring via a written descriptive scale **AND** self-reported scoring via a photo-numeric scale | **Measurement** | **Value** | **p-value** | **Value** | **p-value** | **Value** | **p-value** |
|  | Pearson correlation | 0.216 | 7.15E-13 | 0.227 | 4.81E-14 | 0.198 | 5.51E-11 |
|  | Spearman correlation | 0.155 | 3.09E-07 | 0.171 | 1.43E-08 | 0.147 | 1.00E-06 |
|  | Cohen's Kappa | 0.008 | 1.53E-03 | 0.005 | 4.16E-02 | 0.011 | 3.53E-03 |
|  | Sensitivity (%) | 6.303 |  | 6.154 |  | 6.528 |  |
|  | Specificity (%) | 94.444 |  | 90.244 |  | 95.690 |  |
| Self-reported scoring via a written descriptive scale **OR** self-reported scoring via a photo-numeric scale | **Measurement** | **Value** | **p-value** | **Value** | **p-value** | **Value** | **p-value** |
|  | Pearson correlation | 0.304 | 1.75E-24 | 0.325 | 4.44E-28 | 0.288 | 4.94E-22 |
|  | Spearman correlation | 0.265 | 8.75E-19 | 0.282 | 2.77E-21 | 0.258 | 6.84E-18 |
|  | Cohen's Kappa | 0.057 | 5.89E-16 | 0.048 | 2.11E-14 | 0.074 | 7.46E-15 |
|  | Sensitivity (%) | 39.229 |  | 39.135 |  | 40.518 |  |
|  | Specificity (%) | 77.778 |  | 65.854 |  | 74.138 |  |

**Table S2b**: Exploration of the feasibility of combining photo-numeric scales with descriptive scales (i.e., combinatorial scoring methods) for evaluating eyebags. Here, we explored assessing the photo-numeric scale against combinations of the written scale and assessor-evaluated scoring.

| **Other Standard** | **Measure** | **Putative Gold Standard** | | | | | | | | | | | |
| --- | --- | --- | --- | --- | --- | --- | --- | --- | --- | --- | --- | --- | --- |
|  |  | Self-reported scoring via a written descriptive scale **AND** assessor-evaluated scoring via a photo-numeric scale (Lax definition) | | Self-reported scoring via a written descriptive scale **OR** assessor-evaluated scoring via a photo-numeric scale (Lax definition) | | Self-reported scoring via a written descriptive scale **AND** assessor-evaluated scoring via a photo-numeric scale (Moderately-strict definition) | | Self-reported scoring via a written descriptive scale **OR** assessor-evaluated scoring via a photo-numeric scale (Moderately-strict definition) | | Self-reported scoring via a written descriptive scale **AND** assessor-evaluated scoring via a photo-numeric scale (Strict definition) | | Self-reported scoring via a written descriptive scale **OR** assessor-evaluated scoring via a photo-numeric scale (Strict definition) | |
| Self-reported scoring via a photo-numeric scale | **Measurement** | **Value** | **p-value** | **Value** | **p-value** | **Value** | **p-value** | **Value** | **p-value** | **Value** | **p-value** | **Value** | **p-value** |
|  | Pearson correlation | 0.274 | 4.01E-20 | 0.356 | 1.30E-33 | 0.294 | 6.13E-23 | 0.377 | 6.27E-38 | 0.274 | 4.01E-20 | 0.356 | 1.30E-33 |
|  | Spearman correlation | 0.219 | 2.93E-13 | 0.308 | 3.14E-25 | 0.225 | 7.77E-14 | 0.328 | 1.45E-28 | 0.219 | 2.93E-13 | 0.308 | 3.14E-25 |
|  | Cohen's Kappa | 0.019 | 1.07E-04 | 0.061 | 2.63E-13 | 0.013 | 5.23E-04 | 0.068 | 9.82E-17 | 0.019 | 1.07E-04 | 0.061 | 2.63E-13 |
|  | Sensitivity (%) | 95.238 |  | 88.803 |  | 95.238 |  | 90.112 |  | 95.238 |  | 88.803 |  |
|  | Specificity (%) | 14.447 |  | 15.631 |  | 14.047 |  | 16.038 |  | 14.447 |  | 15.631 |  |

**Table S2c**: Exploration of the feasibility of combining photo-numeric scales with descriptive scales (i.e., combinatorial scoring methods) for evaluating eyebags. Here, we explored assessing the written scale against combinations of the photo-numeric scale and assessor-evaluated scoring.

| **Other Standard** | **Measure** | **Putative Gold Standard** | | | | | | | | | | | |
| --- | --- | --- | --- | --- | --- | --- | --- | --- | --- | --- | --- | --- | --- |
|  |  | Self-reported scoring via a photo-numeric scale **AND** assessor-evaluated scoring via a photo-numeric scale (Lax definition) | | Self-reported scoring via a photo-numeric scale **OR** assessor-evaluated scoring via a photo-numeric scale (Lax definition) | | Self-reported scoring via a photo-numeric scale **AND** assessor-evaluated scoring via a photo-numeric scale (Moderately-strict definition) | | Self-reported scoring via a photo-numeric scale **OR** assessor-evaluated scoring via a photo-numeric scale (Moderately-strict definition) | | Self-reported scoring via a photo-numeric scale **AND** assessor-evaluated scoring via a photo-numeric scale (Strict definition) | | Self-reported scoring via a photo-numeric scale **OR** assessor-evaluated scoring via a photo-numeric scale (Strict definition) | |
| Self-reported scoring via a written descriptive scale | **Measurement** | **Value** | **p-value** | **Value** | **p-value** | **Value** | **p-value** | **Value** | **p-value** | **Value** | **p-value** | **Value** | **p-value** |
|  | Pearson correlation | 0.049 | 1.10E-01 | 0.162 | 8.82E-08 | 0.039 | 2.03E-01 | 0.154 | 3.83E-07 | 0.049 | 1.10E-01 | 0.161 | 1.04E-07 |
|  | Spearman correlation | 0.038 | 2.11E-01 | 0.171 | 1.54E-08 | 0.029 | 3.43E-01 | 0.163 | 7.24E-08 | 0.038 | 2.11E-01 | 0.164 | 5.57E-08 |
|  | Cohen's Kappa | 0.012 | 2.84E-01 | -0.028 | 7.00E-06 | 0.009 | 4.35E-01 | -0.032 | 7.00E-06 | 0.012 | 2.84E-01 | -0.016 | 1.53E-02 |
|  | Sensitivity (%) | 33.636 |  | 29.291 |  | 32.653 |  | 29.390 |  | 33.636 |  | 29.507 |  |
|  | Specificity (%) | 71.267 |  | 77.778 |  | 71.109 |  | 81.250 |  | 71.267 |  | 81.481 |  |
