# Supplementary material for: Comparisons between eyebags, droopy eyelids, and eyebrow positioning identified by photo‐numeric scales or identified by written descriptive scales: Insights from the Singapore/Malaysia cross‐sectional genetics epidemiology study (SMCGES) cohort
Source: Skin Res Technol. 2024 Feb 20;30(2):e13620. doi: 10.1111/srt.13620 (PMC10878178; doi:10.1111/srt.13620)
Supplement: Supplementary file 4 — Supporting Information [file SRT-30-e13620-s002.docx]

**Table S3a**: Exploration of the feasibility of combining photo-numeric scales with descriptive scales (i.e., combinatorial scoring methods) for evaluating droopy eyelids. Here, we explored combining the written and photo-numeric scale together and assessed this against the assessor-evaluated scoring.

| **Other Standard** | **Measure** | **Putative Gold Standard** | | | | | |
| --- | --- | --- | --- | --- | --- | --- | --- |
|  |  | Assessor-evaluated scoring via a photo-numeric scale (Lax definition) | | Assessor-evaluated scoring via a photo-numeric scale (Moderately-strict definition) | | Assessor-evaluated scoring via a photo-numeric scale (Strict definition) | |
| Self-reported scoring via a written descriptive scale **AND** self-reported scoring via a photo-numeric scale | **Measurement** | **Value** | **p-value** | **Value** | **p-value** | **Value** | **p-value** |
|  | Pearson correlation | 0.048 | 1.18E-01 | 0.089 | 3.57E-03 | 0.059 | 5.15E-02 |
|  | Spearman correlation | 0.033 | 2.82E-01 | 0.068 | 2.63E-02 | 0.033 | 2.72E-01 |
|  | Cohen's Kappa | 0.008 | 5.62E-01 | 0.030 | 1.14E-01 | 0.006 | 7.51E-01 |
|  | Sensitivity (%) | 1.587 |  | 3.279 |  | 2.222 |  |
|  | Specificity (%) | 99.267 |  | 99.314 |  | 99.228 |  |
| Self-reported scoring via a written descriptive scale **OR** self-reported scoring via a photo-numeric scale | **Measurement** | **Value** | **p-value** | **Value** | **p-value** | **Value** | **p-value** |
|  | Pearson correlation | 0.252 | 4.10E-17 | 0.303 | 2.33E-24 | 0.248 | 1.26E-16 |
|  | Spearman correlation | 0.234 | 6.31E-15 | 0.296 | 2.35E-23 | 0.231 | 1.38E-14 |
|  | Cohen's Kappa | 0.211 | 2.89E-14 | 0.238 | 2.71E-22 | 0.158 | 2.61E-13 |
|  | Sensitivity (%) | 37.302 |  | 57.377 |  | 53.333 |  |
|  | Specificity (%) | 88.168 |  | 87.745 |  | 86.873 |  |

**Table S3b**: Exploration of the feasibility of combining photo-numeric scales with descriptive scales (i.e., combinatorial scoring methods) for evaluating droopy eyelids. Here, we explored assessing the photo-numeric scale against combinations of the written scale and assessor-evaluated scoring.

| **Other Standard** | **Measure** | **Putative Gold Standard** | | | | | | | | | | | |
| --- | --- | --- | --- | --- | --- | --- | --- | --- | --- | --- | --- | --- | --- |
|  |  | Self-reported scoring via a written descriptive scale **AND** assessor-evaluated scoring via a photo-numeric scale (Lax definition) | | Self-reported scoring via a written descriptive scale **OR** assessor-evaluated scoring via a photo-numeric scale (Lax definition) | | Self-reported scoring via a written descriptive scale **AND** assessor-evaluated scoring via a photo-numeric scale (Moderately-strict definition) | | Self-reported scoring via a written descriptive scale **OR** assessor-evaluated scoring via a photo-numeric scale (Moderately-strict definition) | | Self-reported scoring via a written descriptive scale **AND** assessor-evaluated scoring via a photo-numeric scale (Strict definition) | | Self-reported scoring via a written descriptive scale **OR** assessor-evaluated scoring via a photo-numeric scale (Strict definition) | |
| Self-reported scoring via a photo-numeric scale | **Measurement** | **Value** | **p-value** | **Value** | **p-value** | **Value** | **p-value** | **Value** | **p-value** | **Value** | **p-value** | **Value** | **p-value** |
|  | Pearson correlation | 0.041 | 1.83E-01 | 0.259 | 5.07E-18 | 0.073 | 1.60E-02 | 0.289 | 3.27E-22 | 0.056 | 6.41E-02 | 0.241 | 8.75E-16 |
|  | Spearman correlation | 0.035 | 2.53E-01 | 0.242 | 6.78E-16 | 0.065 | 3.21E-02 | 0.274 | 5.25E-20 | 0.039 | 1.99E-01 | 0.212 | 1.77E-12 |
|  | Cohen's Kappa | 0.011 | 4.03E-01 | 0.217 | 2.35E-15 | 0.018 | 9.11E-02 | 0.252 | 8.68E-19 | 0.004 | 5.92E-01 | 0.184 | 6.36E-11 |
|  | Sensitivity (%) | 22.222 |  | 27.168 |  | 40.000 |  | 34.821 |  | 33.333 |  | 30.612 |  |
|  | Specificity (%) | 89.646 |  | 92.731 |  | 89.684 |  | 92.363 |  | 89.610 |  | 91.556 |  |

**Table S3c**: Exploration of the feasibility of combining photo-numeric scales with descriptive scales (i.e., combinatorial scoring methods) for evaluating droopy eyelids. Here, we explored assessing the written scale against combinations of the photo-numeric scale and assessor-evaluated scoring.

| **Other Standard** | **Measure** | **Putative Gold Standard** | | | | | | | | | | | |
| --- | --- | --- | --- | --- | --- | --- | --- | --- | --- | --- | --- | --- | --- |
|  |  | Self-reported scoring via a photo-numeric scale **AND** assessor-evaluated scoring via a photo-numeric scale (Lax definition) | | Self-reported scoring via a photo-numeric scale **OR** assessor-evaluated scoring via a photo-numeric scale (Lax definition) | | Self-reported scoring via a photo-numeric scale **AND** assessor-evaluated scoring via a photo-numeric scale (Moderately-strict definition) | | Self-reported scoring via a photo-numeric scale **OR** assessor-evaluated scoring via a photo-numeric scale (Moderately-strict definition) | | Self-reported scoring via a photo-numeric scale **AND** assessor-evaluated scoring via a photo-numeric scale (Strict definition) | | Self-reported scoring via a photo-numeric scale **OR** assessor-evaluated scoring via a photo-numeric scale (Strict definition) | |
| Self-reported scoring via a written descriptive scale | **Measurement** | **Value** | **p-value** | **Value** | **p-value** | **Value** | **p-value** | **Value** | **p-value** | **Value** | **p-value** | **Value** | **p-value** |
|  | Pearson correlation | 0.002 | 9.43E-01 | 0.045 | 1.42E-01 | 0.013 | 6.76E-01 | 0.041 | 1.73E-01 | 0.002 | 9.38E-01 | 0.032 | 2.88E-01 |
|  | Spearman correlation | -0.001 | 9.68E-01 | 0.058 | 5.46E-02 | 0.009 | 7.73E-01 | 0.055 | 6.98E-02 | -0.004 | 9.01E-01 | 0.047 | 1.19E-01 |
|  | Cohen's Kappa | -0.007 | 7.87E-01 | 0.022 | 1.53E-01 | 0.001 | 9.83E-01 | 0.021 | 2.33E-01 | -0.011 | 6.22E-01 | 0.015 | 3.68E-01 |
|  | Sensitivity (%) | 5.000 |  | 8.040 |  | 6.250 |  | 8.451 |  | 4.545 |  | 8.088 |  |
|  | Specificity (%) | 94.813 |  | 95.465 |  | 94.852 |  | 95.314 |  | 94.806 |  | 95.238 |  |
