# Supplementary material for: Comparisons between eyebags, droopy eyelids, and eyebrow positioning identified by photo‐numeric scales or identified by written descriptive scales: Insights from the Singapore/Malaysia cross‐sectional genetics epidemiology study (SMCGES) cohort
Source: Skin Res Technol. 2024 Feb 20;30(2):e13620. doi: 10.1111/srt.13620 (PMC10878178; doi:10.1111/srt.13620)
Supplement: Supplementary file 5 — Supporting Information [file SRT-30-e13620-s001.docx]

**Table S4a**: Exploration of the feasibility of combining photo-numeric scales with descriptive scales (i.e., combinatorial scoring methods) for evaluating low eyebrow positioning. Here, we explored combining the written and photo-numeric scale together and assessed this against the assessor-evaluated scoring.

| **Other Standard** | **Measure** | **Putative Gold Standard** | | | | | |
| --- | --- | --- | --- | --- | --- | --- | --- |
|  |  | Assessor-evaluated scoring via a photo-numeric scale (Lax definition) | | Assessor-evaluated scoring via a photo-numeric scale (Moderately-strict definition) | | Assessor-evaluated scoring via a photo-numeric scale (Strict definition) | |
| Self-reported scoring via a written descriptive scale **AND** self-reported scoring via a photo-numeric scale | **Measurement** | **Value** | **p-value** | **Value** | **p-value** | **Value** | **p-value** |
|  | Pearson correlation | 0.035 | 2.52E-01 | 0.046 | 1.28E-01 | 0.025 | 4.04E-01 |
|  | Spearman correlation | 0.030 | 3.27E-01 | 0.031 | 3.15E-01 | 0.015 | 6.16E-01 |
|  | Cohen's Kappa | 0.000 | 7.55E-01 | 0.001 | 6.37E-01 | -0.002 | 4.81E-01 |
|  | Sensitivity (%) | 0.532 |  | 0.605 |  | 0.543 |  |
|  | Specificity (%) | 99.296 |  | 99.608 |  | 99.420 |  |
| Self-reported scoring via a written descriptive scale **OR** self-reported scoring via a photo-numeric scale | **Measurement** | **Value** | **p-value** | **Value** | **p-value** | **Value** | **p-value** |
|  | Pearson correlation | 0.215 | 9.56E-13 | 0.290 | 2.36E-22 | 0.199 | 3.93E-11 |
|  | Spearman correlation | 0.210 | 3.46E-12 | 0.288 | 4.76E-22 | 0.190 | 3.08E-10 |
|  | Cohen's Kappa | 0.064 | 2.32E-12 | 0.101 | 2.11E-23 | 0.089 | 2.06E-11 |
|  | Sensitivity (%) | 21.406 |  | 23.123 |  | 22.962 |  |
|  | Specificity (%) | 90.845 |  | 90.980 |  | 86.957 |  |

**Table S4b**: Exploration of the feasibility of combining photo-numeric scales with descriptive scales (i.e., combinatorial scoring methods) for evaluating low eyebrow positioning. Here, we explored assessing the photo-numeric scale against combinations of the written scale and assessor-evaluated scoring.

| **Other Standard** | **Measure** | **Putative Gold Standard** | | | | | | | | | | | |
| --- | --- | --- | --- | --- | --- | --- | --- | --- | --- | --- | --- | --- | --- |
|  |  | Self-reported scoring via a written descriptive scale **AND** assessor-evaluated scoring via a photo-numeric scale (Lax definition) | | Self-reported scoring via a written descriptive scale **OR** assessor-evaluated scoring via a photo-numeric scale (Lax definition) | | Self-reported scoring via a written descriptive scale **AND** assessor-evaluated scoring via a photo-numeric scale (Moderately-strict definition) | | Self-reported scoring via a written descriptive scale **OR** assessor-evaluated scoring via a photo-numeric scale (Moderately-strict definition) | | Self-reported scoring via a written descriptive scale **AND** assessor-evaluated scoring via a photo-numeric scale (Strict definition) | | Self-reported scoring via a written descriptive scale **OR** assessor-evaluated scoring via a photo-numeric scale (Strict definition) | |
| Self-reported scoring via a photo-numeric scale | **Measurement** | **Value** | **p-value** | **Value** | **p-value** | **Value** | **p-value** | **Value** | **p-value** | **Value** | **p-value** | **Value** | **p-value** |
|  | Pearson correlation | 0.044 | 1.44E-01 | 0.222 | 1.71E-13 | 0.078 | 1.06E-02 | 0.304 | 1.60E-24 | 0.035 | 2.57E-01 | 0.217 | 5.71E-13 |
|  | Spearman correlation | 0.026 | 3.96E-01 | 0.198 | 5.51E-11 | 0.048 | 1.18E-01 | 0.271 | 1.37E-19 | 0.005 | 8.66E-01 | 0.191 | 2.36E-10 |
|  | Cohen's Kappa | 0.001 | 8.31E-01 | 0.107 | 1.51E-12 | 0.003 | 3.20E-01 | 0.161 | 5.85E-26 | -0.003 | 4.61E-01 | 0.108 | 1.11E-12 |
|  | Sensitivity (%) | 57.143 |  | 58.588 |  | 66.667 |  | 65.868 |  | 45.455 |  | 58.592 |  |
|  | Specificity (%) | 48.828 |  | 55.655 |  | 48.837 |  | 55.288 |  | 48.692 |  | 54.682 |  |

**Table S4c**: Exploration of the feasibility of combining photo-numeric scales with descriptive scales (i.e., combinatorial scoring methods) for evaluating low eyebrow positioning. Here, we explored assessing the written scale against combinations of the photo-numeric scale and assessor-evaluated scoring.

| **Other Standard** | **Measure** | **Putative Gold Standard** | | | | | | | | | | | |
| --- | --- | --- | --- | --- | --- | --- | --- | --- | --- | --- | --- | --- | --- |
|  |  | Self-reported scoring via a photo-numeric scale **AND** assessor-evaluated scoring via a photo-numeric scale (Lax definition) | | Self-reported scoring via a photo-numeric scale **OR** assessor-evaluated scoring via a photo-numeric scale (Lax definition) | | Self-reported scoring via a photo-numeric scale **AND** assessor-evaluated scoring via a photo-numeric scale (Moderately-strict definition) | | Self-reported scoring via a photo-numeric scale **OR** assessor-evaluated scoring via a photo-numeric scale (Moderately-strict definition) | | Self-reported scoring via a photo-numeric scale **AND** assessor-evaluated scoring via a photo-numeric scale (Strict definition) | | Self-reported scoring via a photo-numeric scale **OR** assessor-evaluated scoring via a photo-numeric scale (Strict definition) | |
| Self-reported scoring via a written descriptive scale | **Measurement** | **Value** | **p-value** | **Value** | **p-value** | **Value** | **p-value** | **Value** | **p-value** | **Value** | **p-value** | **Value** | **p-value** |
|  | Pearson correlation | 0.032 | 2.95E-01 | 0.072 | 1.84E-02 | 0.020 | 5.08E-01 | 0.051 | 9.26E-02 | 0.019 | 5.31E-01 | 0.056 | 6.70E-02 |
|  | Spearman correlation | 0.023 | 4.50E-01 | 0.059 | 5.09E-02 | 0.009 | 7.59E-01 | 0.042 | 1.68E-01 | 0.008 | 7.98E-01 | 0.044 | 1.48E-01 |
|  | Cohen's Kappa | 0.006 | 5.31E-01 | 0.004 | 2.12E-01 | 0.002 | 8.37E-01 | 0.005 | 2.17E-01 | 0.001 | 9.19E-01 | 0.006 | 1.41E-01 |
|  | Sensitivity (%) | 2.963 |  | 2.449 |  | 2.479 |  | 2.596 |  | 2.344 |  | 2.678 |  |
|  | Specificity (%) | 97.886 |  | 100.000 |  | 97.813 |  | 99.487 |  | 97.796 |  | 99.550 |  |
